# The distance-profile representation and its application to detection of distantly related protein families

**DOI:** 10.1186/1471-2105-6-282

**Published:** 2005-11-29

**Authors:** Chin-Jen Ku, Golan Yona

**Affiliations:** 1 Department of Computer Science, Cornell University, Ithaca, NY, USA

## Abstract

**Background:**

Detecting homology between remotely related protein families is an important problem in computational biology since the biological properties of uncharacterized proteins can often be inferred from those of homologous proteins. Many existing approaches address this problem by measuring the similarity between proteins through sequence or structural alignment. However, these methods do not exploit collective aspects of the protein space and the computed scores are often noisy and frequently fail to recognize distantly related protein families.

**Results:**

We describe an algorithm that improves over the state of the art in homology detection by utilizing global information on the proximity of entities in the protein space. Our method relies on a vectorial representation of proteins and protein families and uses structure-specific association measures between proteins and template structures to form a high-dimensional feature vector for each query protein. These vectors are then processed and transformed to sparse feature vectors that are treated as statistical fingerprints of the query proteins. The new representation induces a new metric between proteins measured by the statistical difference between their corresponding probability distributions.

**Conclusion:**

Using several performance measures we show that the new tool considerably improves the performance in recognizing distant homologies compared to existing approaches such as PSIBLAST and FUGUE.

## Background

The ongoing sequencing efforts continue to discover the sequences of many new proteins, whose function is unknown. Currently, protein databases contain the sequences of about 1,800,000 proteins, of which more than half are partially or completely uncharacterized [1]. Typically, proteins are analyzed by searching for homologous proteins that have already been characterized. Homology establishes the evolutionary relationship among different organisms, and the biological properties of uncharacterized proteins can often be inferred from those of homologous proteins. However, detecting homology between proteins can be a difficult task.

Our ability to detect subtle similarities between proteins depends strongly on the representations we employ for proteins. Sequence and structure are two possible representations of proteins that hinge directly on molecular information. The essential difference between the representation of a protein as a sequence of amino acids and its representation as a 3D structure traditionally dictated different methodologies, different similarity or distance measures and different comparison algorithms. The power of these representations in detecting remote homologies differ markedly. Despite extensive efforts, current methods for sequence analysis often fail to detect remote homologies for sequences that have diverged greatly. In contrast, structure is often conserved more than sequence [2-4], and detecting structural similarity may help infer function beyond what is possible with sequence analysis. However, structural information is sparse and available for only a small part of the protein space.

One may argue that the weakness of sequence based methods is rooted in the underlying representation of proteins, the model used for comparison and/or the comparison algorithm, since in principle, according to the central dogma of molecular biology, (almost) all the information that is needed to form the 3D structure is encoded in the sequence. Indeed, in recent years better sequence-based methods were developed [5-8]. These methods utilize the information in groups of related sequences (a protein or domain family) to build specific statistical models associated to different groups of proteins (*i.e. generative models*) that can be used to search and detect subtle similarities with remotely related proteins. Such generative models assume a statistical source that generates instances according to some underlying distributions, and model the process that generates samples and the corresponding distributions.

When seeking a new similarity measure for proteins that departs from sequence and structure, a natural question is what is the "correct" encoding of proteins? Several works studied the mathematical representation of protein sequences based on sequence properties such as amino acid composition or chemical features [9-12]. However, these representations had limited success, since they did not capture the essence of proteins as ordered sequences of amino acids.

Recently, alternative representations of protein sequences based on the so-called *kernel *methods were proposed. These methods are drawn from the field of machine learning and strive to find an adequate mapping of the protein space onto the Euclidean space where classification techniques such as support vector machines (SVM) or artificial neural networks (ANN) can be applied. Under the kernel representation, each protein is typically mapped to a vector in a (high-dimensional) *feature space*, and the resulting vector is termed *feature vector*. Subsequently, an inner product is defined in the feature space in order to estimate the (dis)similarity among different proteins. A major advantage of the kernel methods is that with an adequate choice of the kernel function, the feature vectors need not be computed explicitly in order to evaluate the similarity relationships. In addition, the users may build a specific feature space such that the kernel function directly estimates these relationships. However, string kernels do not easily lend themselves to this property, and therefore they need to be computed explicitly. The main difference between the different kernel methods reside in the definition of feature elements which are either related to the parameters of some generative process for each group of related proteins or some measure of similarity among the protein sequences.

For instance, the SVM-Fisher algorithm [13] uses the *Fisher kernel *which is based on hidden Markov models (HMMs). The components of the feature vector are the derivatives of the log-likelihood score of the sequence with respect to the parameters of a HMM that has been trained for a particular protein family. Tsuda *et. al. *[14] implemented another representation based on *marginalized *and *joint kernels *and showed that the Fisher kernel is in fact a special case of marginalized kernel. They also experimented with the marginalized count kernels of different orders, that are similar to the *spectrum kernel *which was first introduced by [15]. The spectrum kernel is evaluated by counting the number of times each possible *k*-long subsequence of amino acids (*k*-mer) occurs in one given protein. The marginalized count kernel takes into account both the observed frequency of different subsequences and the context (e.g. exon or intron for DNA sequences). The *mismatch-spectrum kernel *[16] is a generalization of the spectrum kernel that considers also mutation probabilities between *k*-mers which differ by no more than *m *characters. The *homology kernel *[17] is another biologically motivated sequence embedding process that measures the similarity between two proteins by taking into account their respective groups of homologous sequences. It can be thought of as an extension of the mismatch-spectrum kernel by adding a wildcard character and distinguishing the mismatch penalty between two substrings depending on whether the sequences are grouped together or not.

The *covariance kernel *is another type of kernel that uses a framework similar to the one employed by our method. The covariance kernel approach is probabilistic and much work is focused on the implementation of the generative models. In [18], the covariance kernel is based on the *mutual information kernel *which measures the similarity between data samples and a certain generative process. The generative process is characterized by a mediator distribution defined between the (usually vague) prior and the posterior distribution. On the other hand, [19] focuses on the representation of biological sequences using the probabilistic suffix tree [20] as the generative model for different groups of related proteins. The proposed kernel generates a feature vector for protein sequences, where each feature corresponds to a different generative model and its value is the likelihood of the sequence based on that model. Finally, we mention another related work [21] that uses the notion of *pairwise kernel*. Under this framework, each protein is represented by a vector that consists of pairwise sequence similarities with respect to the set of input sequences. It is also worth mentioning the many related studies in the field of natural language processing and text analysis. For example, an approach that in some ways is similar to the pairwise and covariance kernels and in other ways is related to the spectrum kernel approach, is used in [22] to represent verbs and nouns in English texts, with nouns represented as frequency vectors over the set of verbs (based on their association with different verbs) and vice versa. The nouns and verbs are then clustered based on this representation. Studies that attempted to devise automatic methods for text categorization and web-page classification often use similar techniques, where the text is represented as a histogram vector over a vocabulary of words (e.g. [23]).

Here we study a general framework of protein representation called the *distance-profile *representation, that utilizes the global information in the protein space in search of statistical regularities. The representation draws on an association measure between input samples (e.g. proteins and protein families) and can use existing measures of similarity, distance or probability (even if limited to a subset of the input space for which the measure can be applied). This representation induces a new measure of similarity for all protein pairs based on their vectorial representations. Our representation is closely related to the covariance and pairwise kernels described above. However, it is the estimation of pvalues through statistical modeling of the background process, coupled with the transformation to probability distributions, the noise reduction protocols and the choice of the distance function that result in a substantial impact on the performance, and we demonstrate how an adequate choice of the score transformation and the distance metric achieves a considerable improvement in detection of remote homologies.

This paper is organized as follows. We first introduce the notion of distance-profile. We describe how to process the feature vectors through *noise reduction*, and *pvalue transformation *followed by *normalization*. We compare the performance of our new method against several standard algorithms by testing them on a large set of protein families.

## Results

### The distance-profile representation

Our goal is to seek a faithful representation of the protein space that will reflect evolutionary distances even if undetectable by means of existing methods of comparison. We explore a technique based on the distance-profile technique described in [25] and its derivative as applied to protein sequences in [26]. The power of the representation stems from its ability to recover structure in noisy data and boost weak signals [25,26].

The distance-profile representation is simple and can be applied to arbitrary spaces X
 MathType@MTEF@5@5@+=feaafiart1ev1aaatCvAUfKttLearuWrP9MDH5MBPbIqV92AaeXatLxBI9gBamXvP5wqSXMqHnxAJn0BKvguHDwzZbqegm0B1jxALjhiov2DaebbnrfifHhDYfgasaacH8akY=wiFfYdH8Gipec8Eeeu0xXdbba9frFj0=OqFfea0dXdd9vqai=hGuQ8kuc9pgc9s8qqaq=dirpe0xb9q8qiLsFr0=vr0=vr0dc8meaabaqaciaacaGaaeqabaWaaeGaeaaakeaaimaacaWFybaaaa@3973@, Y
 MathType@MTEF@5@5@+=feaafiart1ev1aaatCvAUfKttLearuWrP9MDH5MBPbIqV92AaeXatLxBI9gBamXvP5wqSXMqHnxAJn0BKvguHDwzZbqegm0B1jxALjhiov2DaebbnrfifHhDYfgasaacH8akY=wiFfYdH8Gipec8Eeeu0xXdbba9frFj0=OqFfea0dXdd9vqai=hGuQ8kuc9pgc9s8qqaq=dirpe0xb9q8qiLsFr0=vr0=vr0dc8meaabaqaciaacaGaaeqabaWaaeGaeaaakeaaimaacaWFzbaaaa@3974@ if there exist an **association measure **between instances of X
 MathType@MTEF@5@5@+=feaafiart1ev1aaatCvAUfKttLearuWrP9MDH5MBPbIqV92AaeXatLxBI9gBamXvP5wqSXMqHnxAJn0BKvguHDwzZbqegm0B1jxALjhiov2DaebbnrfifHhDYfgasaacH8akY=wiFfYdH8Gipec8Eeeu0xXdbba9frFj0=OqFfea0dXdd9vqai=hGuQ8kuc9pgc9s8qqaq=dirpe0xb9q8qiLsFr0=vr0=vr0dc8meaabaqaciaacaGaaeqabaWaaeGaeaaakeaaimaacaWFybaaaa@3973@ and instances of Y
 MathType@MTEF@5@5@+=feaafiart1ev1aaatCvAUfKttLearuWrP9MDH5MBPbIqV92AaeXatLxBI9gBamXvP5wqSXMqHnxAJn0BKvguHDwzZbqegm0B1jxALjhiov2DaebbnrfifHhDYfgasaacH8akY=wiFfYdH8Gipec8Eeeu0xXdbba9frFj0=OqFfea0dXdd9vqai=hGuQ8kuc9pgc9s8qqaq=dirpe0xb9q8qiLsFr0=vr0=vr0dc8meaabaqaciaacaGaaeqabaWaaeGaeaaakeaaimaacaWFzbaaaa@3974@ such as a distance function, similarity function or a probability measure. Given an instance *X *in the input space X
 MathType@MTEF@5@5@+=feaafiart1ev1aaatCvAUfKttLearuWrP9MDH5MBPbIqV92AaeXatLxBI9gBamXvP5wqSXMqHnxAJn0BKvguHDwzZbqegm0B1jxALjhiov2DaebbnrfifHhDYfgasaacH8akY=wiFfYdH8Gipec8Eeeu0xXdbba9frFj0=OqFfea0dXdd9vqai=hGuQ8kuc9pgc9s8qqaq=dirpe0xb9q8qiLsFr0=vr0=vr0dc8meaabaqaciaacaGaaeqabaWaaeGaeaaakeaaimaacaWFybaaaa@3973@, a reference set {*Y*_1_, *Y*_2_, ... *Y*_*n*_} of entities in Y
 MathType@MTEF@5@5@+=feaafiart1ev1aaatCvAUfKttLearuWrP9MDH5MBPbIqV92AaeXatLxBI9gBamXvP5wqSXMqHnxAJn0BKvguHDwzZbqegm0B1jxALjhiov2DaebbnrfifHhDYfgasaacH8akY=wiFfYdH8Gipec8Eeeu0xXdbba9frFj0=OqFfea0dXdd9vqai=hGuQ8kuc9pgc9s8qqaq=dirpe0xb9q8qiLsFr0=vr0=vr0dc8meaabaqaciaacaGaaeqabaWaaeGaeaaakeaaimaacaWFzbaaaa@3974@ (e.g. proteins or protein families, sequences or generative models) and an underlying association measure, we associate with the instance *X *a position in a high dimensional space, where every coordinate is associated with one member of the reference set and its value is the similarity with that particular reference object. I.e., we map *X *to a vector of dimension *n *in the host space

X→X=(S (X,Y1)⋮S(X,Yn)).     (1)
 MathType@MTEF@5@5@+=feaafiart1ev1aaatCvAUfKttLearuWrP9MDH5MBPbIqV92AaeXatLxBI9gBaebbnrfifHhDYfgasaacH8akY=wiFfYdH8Gipec8Eeeu0xXdbba9frFj0=OqFfea0dXdd9vqai=hGuQ8kuc9pgc9s8qqaq=dirpe0xb9q8qiLsFr0=vr0=vr0dc8meaabaqaciGacaGaaeqabaqabeGadaaakeaacqWGybawcqGHsgIRtCvAUfeBSjuyZL2yd9gzLbvyNv2CaeHbwvMCKfMBHbaceeGaa8hwaiabg2da9maabmaabaqbaeqabmqaaaqaaiabdofatjaaykW7daqadaqaaiabdIfayjabcYcaSiabdMfaznaaBaaaleaacqaIXaqmaeqaaaGccaGLOaGaayzkaaaabaGaeSO7I0eabaGaem4uam1aaeWaaeaacqWGybawcqGGSaalcqWGzbqwdaWgaaWcbaGaemOBa4gabeaaaOGaayjkaiaawMcaaaaaaiaawIcacaGLPaaacqGGUaGlcaWLjaGaaCzcamaabmaabaGaeGymaedacaGLOaGaayzkaaaaaa@54CA@

This simple representation leads to the definition of a new distance or similarity measure among samples based on their vectorial representation, and when the reference set is identical to the input space (X
 MathType@MTEF@5@5@+=feaafiart1ev1aaatCvAUfKttLearuWrP9MDH5MBPbIqV92AaeXatLxBI9gBamXvP5wqSXMqHnxAJn0BKvguHDwzZbqegm0B1jxALjhiov2DaebbnrfifHhDYfgasaacH8akY=wiFfYdH8Gipec8Eeeu0xXdbba9frFj0=OqFfea0dXdd9vqai=hGuQ8kuc9pgc9s8qqaq=dirpe0xb9q8qiLsFr0=vr0=vr0dc8meaabaqaciaacaGaaeqabaWaaeGaeaaakeaaimaacaWFybaaaa@3973@ = Y
 MathType@MTEF@5@5@+=feaafiart1ev1aaatCvAUfKttLearuWrP9MDH5MBPbIqV92AaeXatLxBI9gBamXvP5wqSXMqHnxAJn0BKvguHDwzZbqegm0B1jxALjhiov2DaebbnrfifHhDYfgasaacH8akY=wiFfYdH8Gipec8Eeeu0xXdbba9frFj0=OqFfea0dXdd9vqai=hGuQ8kuc9pgc9s8qqaq=dirpe0xb9q8qiLsFr0=vr0=vr0dc8meaabaqaciaacaGaaeqabaWaaeGaeaaakeaaimaacaWFzbaaaa@3974@), an iterative application of this representation can be used to form hierarchical clustering over the input samples [25]. In this paper we demonstrate the application of this method to the problem of homology detection between distantly related proteins.

One might observe the resemblance of our method with pairwise kernels and covariance kernels mentioned in the 'Background' section. However, it is the processing of the feature vectors and the choice of the metric, as is laid out next, which are the crucial ingredients that differentiate our method from the previous studies. As exemplified in this paper, under the proper transformations the distance-profile representation has mathematical and statistical interpretations that have other implications, and it is these transformations that deem this method very effective for homology detection, database search and clustering.

### The reference set

Remotely related proteins usually share little sequence similarity, however, they are expected to have similar structures. Therefore, as a reference set for our experiments we chose a non-redundant structure library consisting of domain structures that represent the current protein structure space. The set is derived from the SCOP database [27], release 1.57. Specifically, we used the Genetic Domain Sequence dataset that we downloaded from the Astral webpage [28]. The dataset is obtained from 14,729 entries in the Protein Data Bank (PDB) [29] and contains only protein domains with less than 40% identity between pairs of sequences. Our library consists of 3,964 distinct SCOP domains covering in total 644 folds, 997 superfamilies, and 1,678 families. For notation purpose, we denote this library of template proteins by *SCOP-DB*.

### The association measure

We rely on "structure-aided" sequence alignment to bridge the gap between the sequence space and structure space. Given the library of structures Y
 MathType@MTEF@5@5@+=feaafiart1ev1aaatCvAUfKttLearuWrP9MDH5MBPbIqV92AaeXatLxBI9gBamXvP5wqSXMqHnxAJn0BKvguHDwzZbqegm0B1jxALjhiov2DaebbnrfifHhDYfgasaacH8akY=wiFfYdH8Gipec8Eeeu0xXdbba9frFj0=OqFfea0dXdd9vqai=hGuQ8kuc9pgc9s8qqaq=dirpe0xb9q8qiLsFr0=vr0=vr0dc8meaabaqaciaacaGaaeqabaWaaeGaeaaakeaaimaacaWFzbaaaa@3974@ = {*Y*_1_, *Y*_2_, *Y*_3_, ..., *Y*_*n*_}, every protein sequence *P *is mapped to a *structure-specific n*-dimensional feature vector, as is described above, where the association measure *S*(*P*, *Y*_*i*_) is the similarity score of the sequence-structure alignment between the sequence *P *and the template structure *Y*_*i*_, computed with the FUGUE threading algorithm [30] (see Appendix). From this point on we discuss the application of the distance-profile representation with threading-based association measures. However, we note that the methods described in this paper can be used to process feature vectors using other association measures. In the 'Discussion' section we show that a similar approach applied to sequence-profile alignment scores also produces a considerable improvement in detecting remote homologies.

### Processing feature vectors: pvalue conversion and normalization

The choice of the underlying association function *S*(*P*, *Y*_*i*_) can have a drastic impact on the effectiveness of the representation and we tested several variations.

#### The score association measure

A possible choice is obviously the score reported by the algorithm that compares entities of X
 MathType@MTEF@5@5@+=feaafiart1ev1aaatCvAUfKttLearuWrP9MDH5MBPbIqV92AaeXatLxBI9gBamXvP5wqSXMqHnxAJn0BKvguHDwzZbqegm0B1jxALjhiov2DaebbnrfifHhDYfgasaacH8akY=wiFfYdH8Gipec8Eeeu0xXdbba9frFj0=OqFfea0dXdd9vqai=hGuQ8kuc9pgc9s8qqaq=dirpe0xb9q8qiLsFr0=vr0=vr0dc8meaabaqaciaacaGaaeqabaWaaeGaeaaakeaaimaacaWFybaaaa@3973@ with entities of Y
 MathType@MTEF@5@5@+=feaafiart1ev1aaatCvAUfKttLearuWrP9MDH5MBPbIqV92AaeXatLxBI9gBamXvP5wqSXMqHnxAJn0BKvguHDwzZbqegm0B1jxALjhiov2DaebbnrfifHhDYfgasaacH8akY=wiFfYdH8Gipec8Eeeu0xXdbba9frFj0=OqFfea0dXdd9vqai=hGuQ8kuc9pgc9s8qqaq=dirpe0xb9q8qiLsFr0=vr0=vr0dc8meaabaqaciaacaGaaeqabaWaaeGaeaaakeaaimaacaWFzbaaaa@3974@. (for example, the zscore reported by the FUGUE threading algorithm). We denote feature vectors that are based on the score association measure by **P**^*score*^.

#### The pvalue association measure

If the association measure is distributed over a wide range, the most significant scores will inevitably shadow other numerically less important but still significant matches, thus reducing the sensitivity of the representation. This is the case with most types of similarity scores, including the threading zscores assigned by the FUGUE program.

**Figure 1:**
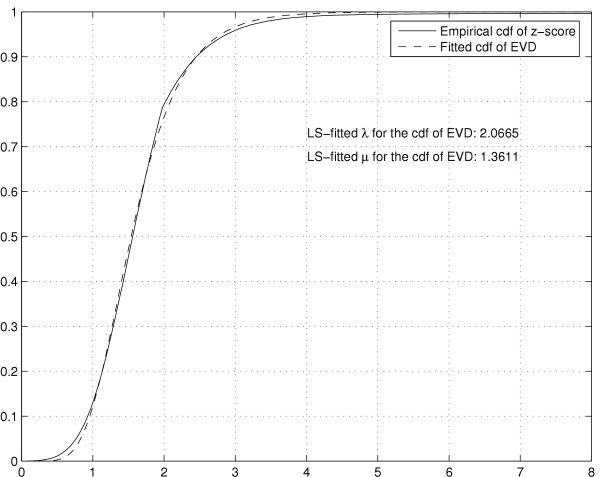
The empirical cdf of the zscores obtained by FUGUE and the fitted EVD cdf with parameters *λ *= 2.0665 and *μ *= 1.3611.

To address this problem we convert the zscores to their underlying cumulative distribution function (cdf) value, where the amplitude of outlier zscores is reduced to within a reasonable range. As was shown in [31], the zscores of local alignment scores follow the extreme value distribution (EVD), see Figure 1, whose cumulative function *F*(*x*) takes the form

*F*(*x*) = *Prob*(*x*' ≤ *x*) = *e*^-*φ*(*x*) ^    *where *   *φ*(*x*) = *e*^-*λ*(*x*-*μ*)^.     (2)

Based on this background distribution we replace the original zscores with a new association measure such that *S*'(*P*, *Y*_*i*_) = *F*(*S*(*P*, *Y*_*i*_)) where *S*(*P*, *X*_*i*_) is the similarity zscore reported by FUGUE. With this transformation, all coordinates are bounded between 0 and 1, with high zscores transformed to values close to 1. Note that the *pvalue *of a given zscore *x *is *pvalue*(*x*) = 1 - *F*(*x*). We denote feature vectors that are based on the *F*(*x*) association measure by **P**^*pvalue*^. It should also be noted that in practice *F*(*x*) = 1 - *pvalue*(*x*) = 1 for large *x *because of machine precision limitations. Therefore, the pvalues that are associated to significant zscores (typically above 5) are approximated by their empirical distribution, thus allowing distinction between a pair of highly significant yet numerically disparate zscores, *e.g. *10 versus 60.

#### The probability association measure

the third variation we tested is based on a simple normalization of each feature vector to form a probability distribution. This transformation enables us to explore distance measures that are suited for probability vectors, as described in section 'Metrics and score functions'. Indeed, in this representation, the normalized vector entries can be considered as the coefficients of a mixture model where the components models are the protein structures, each one inducing a different probability distribution over the protein sequence space. This interpretation emphasizes the similarity with covariance methods which also resort to a probabilistic representation of different protein families as described in the 'Background' section. We denote feature vectors that are based on this association measure by **P**^*prob*^.

### Reducing noise: sparse feature vectors

Our reference set is composed of proteins that belong to different protein families and folds (see section 'The reference set'). Within that data set no two proteins share more than 40% sequence identity. Therefore, for a given query protein we expect to observe only a few significant similarity values in the vector **P**. That is, the entries that correspond to the structural templates of protein families that are related to the query. In other words, the feature vectors contain many entries that are essentially random and meaningless. These random numbers will contribute to the differences between feature vectors, thus masking possibly significant similarities. To reduce noise due to unrelated proteins we eliminate all entries with zscore below a certain threshold *τ*, or *pvalue *above a certain threshold *τ*', to reflect the fact that the corresponding sequence-structure pair is considered irrelevant (Another alternative is to weight the differences by the significance of the measurements. However, to speed up the processing and comparison of feature vectors we adopted the threshold approach.) The parameter *τ*(or *τ*') is optimized to maximize performance, as described in section 'Parameter optimization'. Note that entries with low zscores that are filtered in this step (assigned 0 zscore) remain zero under the transformation to the cdf pvalue as described above. The processed feature vector is denoted by P^score
 MathType@MTEF@5@5@+=feaafiart1ev1aaatCvAUfKttLearuWrP9MDH5MBPbIqV92AaeXatLxBI9gBaebbnrfifHhDYfgasaacH8akY=wiFfYdH8Gipec8Eeeu0xXdbba9frFj0=OqFfea0dXdd9vqai=hGuQ8kuc9pgc9s8qqaq=dirpe0xb9q8qiLsFr0=vr0=vr0dc8meaabaqaciGacaGaaeqabaqabeGadaaakeaatCvAUfeBSjuyZL2yd9gzLbvyNv2CaeHbwvMCKfMBHbaceeGab8huayaajaWaaWbaaSqabeaacqWGZbWCcqWGJbWycqWGVbWBcqWGYbGCcqWGLbqzaaaaaa@3F2D@.

The noise reduction is applied to the original feature vectors and is followed by the pvalue conversion and the normalization to yield new feature vectors **P**^*pvalue *^and **P**^*prob*^, respectively. To illustrate the impact of our procedures on the distance profiles, let us consider the example of two closely related proteins *d*1*qmva_*(*Thioredoxin peroxidase 2*) and *d*1*hd*2*a_ *(*Peroxiredoxin 5*) that belong to family c.47.1.10 under the SCOP denomination. The sequences were threaded against the SCOP library of structural domains, and feature vectors were compiled from the zscores reported by FUGUE. In Table 1 we report the 2163th up to the 2169th entries of their original feature vectors as well as their transformations after noise reduction, pvalue conversion and normalization. As this example demonstrates, the zscore entries are noisy and spread over a wide numerical range. For instance, the 2167th entry of both vectors correspond to their threading score versus the structure of *d*1*prxa_ *(*HorF6 peroxidase*), another protein that is in the same SCOP family c.47.1.10. While the zscore reaches 15.79 for *d*1*qmva_*, it is only 6.38 for *d*1*hd*2*a_*. These large differences will inevitably result in large distances between the feature vectors despite the fact that they have significant zscore values in the same positions. The pvalue conversion and normalization (third and fourth rows in the table) resolve this problem by rescaling scores to within a fixed interval.

**Table 1:** Illustration of noise reduction, pvalue conversion and normalization on the feature vectors associated with proteins *d*1*qmva_ *(*Thioredoxin peroxidase 2*) (denoted by c.47.1.10.3) and *d*1*hd*2*a_ *(*Peroxiredoxin 5*) (denoted by c.47.1.10.4). We display the 2163th up to the 2170th entries of the different feature vectors. The zscore cutoff value *τ*is set at 3.5. The feature vector for *c.47.1.10.3 *reaches its maximum at the 2165th position which corresponds to the self-alignment zscore.

Representation	Sequence	2163th to 2170th entries of the feature vectors							
**P**^score^	*c.47.1.10.3*	0	5.170	63.210	6.420	15.790	4.150	0	1.590
	*c.47.1.10.4*	1.980	1.730	7.070	53.530	6.380	3.290	0	0
P^score MathType@MTEF@5@5@+=feaafiart1ev1aaatCvAUfKttLearuWrP9MDH5MBPbIqV92AaeXatLxBI9gBaebbnrfifHhDYfgasaacH8akY=wiFfYdH8Gipec8Eeeu0xXdbba9frFj0=OqFfea0dXdd9vqai=hGuQ8kuc9pgc9s8qqaq=dirpe0xb9q8qiLsFr0=vr0=vr0dc8meaabaqaciGacaGaaeqabaqabeGadaaakeaatCvAUfeBSjuyZL2yd9gzLbvyNv2CaeHbwvMCKfMBHbaceeGab8huayaajaWaaWbaaSqabeaaieaacqGFZbWCcqGFJbWycqGFVbWBcqGFYbGCcqGFLbqzaaaaaa@3F1D@	*c.47.1.10.3*	0	5.170	63.210	6.420	15.790	4.150	0	0
	*c.47.1.10.4*	0	0	7.070	53.530	6.380	0	0	0
**P**^pvalue^	*c.47.1.10.3*	0	0.998916	0.999921	0.999163	0.999619	0.996863	0	0
	*c.47.1.10.4*	0	0	0.999234	0.999888	0.999158	0	0	0
**P**^prob^	*c.47.1.10.3*	0	0.052803	0.052856	0.052816	0.052840	0.052694	0	0
	*c.47.1.10.4*	0	0	0.077210	0.077260	0.077204	0	0	0

### Metrics and score functions

Under the distance-profile representation, the similarity (distance) between two protein sequences *P *and *P' *is defined as the similarity (distance) of their corresponding feature vectors

*S*(*P*, *P'*) = *f*(**P, P**')

The function *f *can be a similarity function or a distance function and we considered several different variants. We tested the *L*_2 _norm (the Euclidean metric) and the *L*_1 _norm (the Manhattan distance). For probability distributions we also tested the Jensen-Shannon (JS) measure of divergence [34]. Given two probability distributions **p **and **q**, for every 0 ≤ *λ *≤ 1, their *λ*-Jensen-Shannon *divergence *is defined as

DλJS[p||q]=λDKL[p||r]+(1−λ)DKL[q||r]
 MathType@MTEF@5@5@+=feaafiart1ev1aaatCvAUfKttLearuWrP9MDH5MBPbIqV92AaeXatLxBI9gBaebbnrfifHhDYfgasaacH8akY=wiFfYdH8Gipec8Eeeu0xXdbba9frFj0=OqFfea0dXdd9vqai=hGuQ8kuc9pgc9s8qqaq=dirpe0xb9q8qiLsFr0=vr0=vr0dc8meaabaqaciGacaGaaeqabaqabeGadaaakeaacqWGebardaqhaaWcbaGaeq4UdWgabaGaemOsaOKaem4uamfaaOGaei4waS1exLMBbXgBcf2CPn2qVrwzqf2zLnharyGvLjhzH5wyaGabbiaa=bhacqGG8baFcqGG8baFcaWFXbGaeiyxa0Laeyypa0Jaeq4UdWMaemiraq0aaWbaaSqabeaacqWGlbWscqWGmbataaGccqGGBbWwcaWFWbGaeiiFaWNaeiiFaWNaa8NCaiabc2faDjabgUcaRiabcIcaOiabigdaXiabgkHiTiabeU7aSjabcMcaPiabdseaenaaCaaaleqabaGaem4saSKaemitaWeaaOGaei4waSLaa8xCaiabcYha8jabcYha8jaa=jhacqGGDbqxaaa@629D@

where *D*^*KL *^[**p||q**] is the Kullback-Leibler (KL) divergence [35] defined as DKL[p||q]=∑ipilog⁡2piqi
 MathType@MTEF@5@5@+=feaafiart1ev1aaatCvAUfKttLearuWrP9MDH5MBPbIqV92AaeXatLxBI9gBaebbnrfifHhDYfgasaacH8akY=wiFfYdH8Gipec8Eeeu0xXdbba9frFj0=OqFfea0dXdd9vqai=hGuQ8kuc9pgc9s8qqaq=dirpe0xb9q8qiLsFr0=vr0=vr0dc8meaabaqaciGacaGaaeqabaqabeGadaaakeaacqWGebardaahaaWcbeqaaiabdUealjabdYeambaakiabcUfaBnXvP5wqSXMqHnxAJn0BKvguHDwzZbqegyvzYrwyUfgaiqqacaWFWbGaeiiFaWNaeiiFaWNaa8xCaiabc2faDjabg2da9maaqababaGaemiCaa3aaSbaaSqaaiabdMgaPbqabaGccyGGSbaBcqGGVbWBcqGGNbWzdaWgaaWcbaGaeGOmaidabeaakmaalaaabaGaemiCaa3aaSbaaSqaaiabdMgaPbqabaaakeaacqWGXbqCdaWgaaWcbaGaemyAaKgabeaaaaaabaGaemyAaKgabeqdcqGHris5aaaa@549A@ and **r **= *λ***p **+ (1 - *λ*)**q **can be considered as the most likely common source distribution of both distributions **p **and **q**, with *λ *as a prior weight (without *a priori *information, a natural choice is *λ *= 1/2). Unlike the Kullback-Leibler measure, the JS measure is symmetric and bounded. It ranges between 0 and 1, where the divergence for identical distributions is 0. This measure has been used successfully in [7,36,37] to detect subtle similarities between statistical models of protein families and in [38] for automatic domain prediction from sequence information.

**Figure 2:**
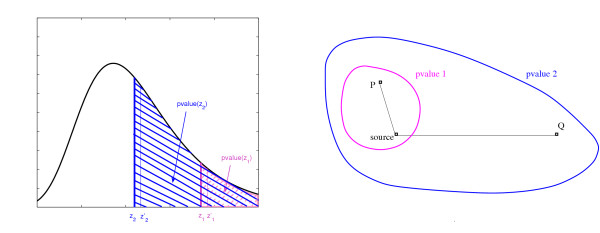
**The pvalue distance**. **Left: **Often is the case that the distance between two measurements depends not only on the relative nominal difference between the measurements but also on the absolute magnitude of the each one of the measurements. For example, two measurements *z*_1 _and  are statistically more similar to each other than the two measurements *z*_2 _and . That is to say that there are fewer measurements with score as high as *z*_1 _and  and therefore fewer instances that have similar properties. The measurements in our case are the zscores that indicate the significance of the match between a sequence and a structural template (the *source*). For a given zscore *z*, the pvalue measure *pvalue*(*z*) is an estimate of the total probability mass in the protein sequence space of sequences that match the structural template with *zscore *≥ *z*. **Right: **The least significant pvalue of the two associated with the two measurements is an estimate of the mass of sequences with similar properties (note *pvalue*1 <*pvalue*2).

As an alternative approach to assess the similarity of a pair of proteins based on their distance-profile representation we propose the **pvalue-distance **(PD) function. This function assesses the distance between two proteins by estimating the probability to observe a random protein with a feature vector inside the volume delimited by their two feature vectors. The smaller the volume is, the more similar are the two vectors. The function operates on the *pvalues *used to form the feature vectors **P**^*pvalue*^. Given two feature vectors **P **and **Q **that correspond to proteins *P *and *Q*, we consider one coordinate *i *at a time and estimate the total probability mass of samples whose *i*-th feature is bounded between the feature values *p*_*i *_and *q*_*i *_as is illustrated in Figure 2a. Since each representative in the reference set induces a complex high-dimensional distribution over the protein sequence space, the one-dimensional pvalue measure *p*_*i *_can only serve to approximate a certain perimeter in the sequence space of sequences that are as similar or more similar to the *i-*th source than the protein *P*. Therefore, we use the least significant *pvalue *of *p*_*i *_and *q*_*i *_as an upper bound estimator of the volume of relevant instances, as illustrated in Figure 2b. The total volume is computed by taking the product over all coordinates.

Formally, consider the two pvalue feature vectors P1pvalue=(p11…pn1)
 MathType@MTEF@5@5@+=feaafiart1ev1aaatCvAUfKttLearuWrP9MDH5MBPbIqV92AaeXatLxBI9gBaebbnrfifHhDYfgasaacH8akY=wiFfYdH8Gipec8Eeeu0xXdbba9frFj0=OqFfea0dXdd9vqai=hGuQ8kuc9pgc9s8qqaq=dirpe0xb9q8qiLsFr0=vr0=vr0dc8meaabaqaciGacaGaaeqabaqabeGadaaakeaaieqacqWFqbaudaqhaaWcbiqaaCQccqWFXaqmaeaacqWFWbaCcqWF2bGDcqWFHbqycqWFSbaBcqWF1bqDcqWFLbqzaaGccqGH9aqpcqGGOaakcqWGWbaCdaqhaaWcbaGaeGymaedabaGaeGymaedaaOGaeSOjGSKaemiCaa3aa0baaSqaaiabd6gaUbqaaiabigdaXaaakiabcMcaPaaa@4336@ and P2pvalue=(p12…pn2)
 MathType@MTEF@5@5@+=feaafiart1ev1aaatCvAUfKttLearuWrP9MDH5MBPbIqV92AaeXatLxBI9gBaebbnrfifHhDYfgasaacH8akY=wiFfYdH8Gipec8Eeeu0xXdbba9frFj0=OqFfea0dXdd9vqai=hGuQ8kuc9pgc9s8qqaq=dirpe0xb9q8qiLsFr0=vr0=vr0dc8meaabaqaciGacaGaaeqabaqabeGadaaakeaaieqacqWFqbaudaqhaaWcbaGae8NmaidabaGae8hCaaNae8NDayNae8xyaeMae8hBaWMae8xDauNae8xzaugaaOGaeyypa0JaeiikaGIaemiCaa3aa0baaSqaaiabigdaXaqaaiabikdaYaaakiablAciljabdchaWnaaDaaaleaacqWGUbGBaeaacqaIYaGmaaGccqGGPaqkaaa@4288@ that are obtained by mapping each zscore *z*_*i *_to its pvalue *p*_*i *_using the EVD background distribution as described in section 'Processing feature vectors'. Their pvalue distance is defined as

PD(P1,P2)=∏i=1Lmax⁡(pi1,pi2).     (3)
MathType@MTEF@5@5@+=feaafiart1ev1aaatCvAUfKttLearuWrP9MDH5MBPbIqV92AaeXatLxBI9gBaebbnrfifHhDYfgasaacH8akY=wiFfYdH8Gipec8Eeeu0xXdbba9frFj0=OqFfea0dXdd9vqai=hGuQ8kuc9pgc9s8qqaq=dirpe0xb9q8qiLsFr0=vr0=vr0dc8meaabaqaciGacaGaaeqabaqabeGadaaakeaacqWGqbaucqWGebarcqGGOaaktCvAUfeBSjuyZL2yd9gzLbvyNv2CaeHbwvMCKfMBHbaceeGaa8huamaaBaaaleaacaWFXaaabeaakiabcYcaSiaa=bfadaWgaaWcbaGaa8NmaaqabaGccqGGPaqkcqGH9aqpdaqeWaqaaiGbc2gaTjabcggaHjabcIha4naabmaabaGaemiCaa3aa0baaSqaaiabdMgaPbqaaiabigdaXaaakiabcYcaSiabdchaWnaaDaaaleaacqWGPbqAaeaacqaIYaGmaaaakiaawIcacaGLPaaaaSqaaiabdMgaPjabg2da9iabigdaXaqaaiabdYeambqdcqGHpis1aOGaeiOla4IaaCzcaiaaxMaadaqadaqaaiabiodaZaGaayjkaiaawMcaaaaa@59F8@

In practice, the PD score is evaluated through its logarithm:

−log⁡PD(P1,P2)=−∑i=1Llog⁡max⁡(pi1,pi2)     (4)
 MathType@MTEF@5@5@+=feaafiart1ev1aaatCvAUfKttLearuWrP9MDH5MBPbIqV92AaeXatLxBI9gBaebbnrfifHhDYfgasaacH8akY=wiFfYdH8Gipec8Eeeu0xXdbba9frFj0=OqFfea0dXdd9vqai=hGuQ8kuc9pgc9s8qqaq=dirpe0xb9q8qiLsFr0=vr0=vr0dc8meaabaqaciGacaGaaeqabaqabeGadaaakeaacqGHsislcyGGSbaBcqGGVbWBcqGGNbWzcqWGqbaucqWGebarcqGGOaaktCvAUfeBSjuyZL2yd9gzLbvyNv2CaeHbwvMCKfMBHbaceeGaa8huamaaBaaaleaacaWFXaaabeaakiabcYcaSiaa=bfadaWgaaWcbaGaa8NmaaqabaGccqGGPaqkcqGH9aqpcqGHsisldaaeWaqaaiGbcYgaSjabc+gaVjabcEgaNjGbc2gaTjabcggaHjabcIha4bWcbaGaemyAaKMaeyypa0JaeGymaedabaGaemitaWeaniabggHiLdGcdaqadaqaaiabdchaWnaaDaaaleaacqWGPbqAaeaacqaIXaqmaaGccqGGSaalcqWGWbaCdaqhaaWcbaGaemyAaKgabaGaeGOmaidaaaGccaGLOaGaayzkaaGaaCzcaiaaxMaadaqadaqaaiabisda0aGaayjkaiaawMcaaaaa@633D@

(It should be noted that here the measure *pvalue*(*z*) is used directly to define the feature values, as opposed to *F*(*z*) = 1 - *pvalue*(*z*) that was used before when compiling the feature vectors **P**^*pvalue*^. However, to simplify notation we also refer to these feature vectors as **P**^*pvalue*.)^

To distinguish all the measures discussed in this section from the association measures discussed in section 'Processing feature vectors', we refer to all of them from now on as distance metrics, although they are not necessarily metrics or distance functions.

## Discussion

### Dataset preparation

We use the SCOP classification of protein structures [27] as our benchmark. The SCOP database is built mostly based on manual analysis of protein structures and is characterized by a hierarchy with four main levels: class, fold, superfamily and family. Proteins that belong to the same family display significant sequence similarity that indicates homology. At the next level (superfamily), families are grouped into superfamilies based on structural similarity and weak sequence similarity (e.g. conserved functional residues). Proteins that belong to different families within the same superfamily are considered remotely related. It is this level that has been used in many studies to evaluate sequence comparison algorithms (e.g. [7,39,40]). The challenge is to automatically detect similarities between families within the same superfamily, that were established manually by the SCOP experts.

To determine the optimal parameters for the distance-profile representation and compare its performance to other algorithms, we split the library *SCOP-DB *into a training set and a test set. Since our purpose is to test the ability to find remotely related proteins at the superfamily and fold levels, we first discard all proteins that have fewer than 5 remote homologs in *SCOP-DB *(i.e. are in superfamilies of size 5 or less). From the remaining 2,570 sequences we randomly select 100 for the training set, and the rest (2,470 sequences) are compiled into the test set.

### Performance indices

To evaluate the performance of a given method for a specific query protein we compare the protein against *SCOP-DB*, sort the results and assess the correlation of the sorted list with established homology relations. In our experiments we consider two proteins to be related (a positive pair) if they belong to the same SCOP superfamily. All other pairs are treated as negatives. We also consider a more relaxed definition, where proteins are deemed related if they belong to the same SCOP fold.

A popular measure of performance used in signal detection and classification is the ROC*k *measure [41]. This is the cumulative count of positive samples detected until *k *negative samples are met in the sorted list of results. We use four different indices to assess performance, all are variations on commonly used sensitivity and accuracy measures. These indices measure the ability of a given algorithm to recognize different levels of structural similarity between protein sequences and within neighborhoods of varying sizes:

• The ROC1 superfamily index **(ROC1-S)**.

• The ROC1-fold index **(ROC1-F)**.

• The top-superfamily-superfamily index **(TSS)**.

• The top-fold-fold index **(TFF)**.

Given the sorted list of results for a query protein *p*, the ROC1-S index totals the number of proteins in the same *superfamily *as *p *that are observed from the top of the sorted list until the first false match (*i.e. *different superfamily) appears. Likewise, the ROC1-F index is defined by counting the number of proteins in the same *fold *as *p *from the top of the sorted list until the first match that involves two proteins with different folds. The last two indices are characterized by the following generic definition: the *top-X-Y *index for a protein *p *counts the total number of proteins sharing the same *Y *SCOP denomination among the *n*_*X *_closest sequences of *p*, where *n*_*X *_is the total number of sequences in the library that have the same *X *SCOP denomination as *p *itself (For example, to compute the top-fold-fold index for a query protein that belongs to a SCOP fold containing *n *proteins, we look at the top *n *proteins in the sorted list and count how many of them are actually in the same fold as the query protein). Self-similarity is ignored in the assessment of all performance indices.

Note that all these indices are closely related to sensitivity measures at different levels, however, the relevant neighborhood is calibrated on a per-superfamily/fold basis. And while the two ROC indices stop as soon as one false match is encountered, the top-*X-Y *indices credit a method that detects many true positives at the top even if mixed with a few false positives. Therefore, a method yielding a lower ROC1 index but higher TSS (or TFF) should be still considered successful since it clusters the query close to a larger number of related objects.

To obtain the overall performance of a method with respect to a set of queries, we simply take the sum of all their corresponding performance indices as the global result. I.e. given a query set **Q **= {*q*_1_, ..., *q*_*n*_}, the performance of a method *M*, using index *I *is

I(M,Q)=∑i=1nI(M,qi)
 MathType@MTEF@5@5@+=feaafiart1ev1aaatCvAUfKttLearuWrP9MDH5MBPbIqV92AaeXatLxBI9gBaebbnrfifHhDYfgasaacH8akY=wiFfYdH8Gipec8Eeeu0xXdbba9frFj0=OqFfea0dXdd9vqai=hGuQ8kuc9pgc9s8qqaq=dirpe0xb9q8qiLsFr0=vr0=vr0dc8meaabaqaciGacaGaaeqabaqabeGadaaakeaacqWGjbqscqGGOaakcqWGnbqtcqGGSaaltCvAUfeBSjuyZL2yd9gzLbvyNv2CaeHbwvMCKfMBHbaceeGaa8xuaiabcMcaPiabg2da9maaqahabaGaemysaKKaeiikaGIaemyta0KaeiilaWIaemyCae3aaSbaaSqaaiabdMgaPbqabaGccqGGPaqkaSqaaiabdMgaPjabg2da9iabigdaXaqaaiabd6gaUbqdcqGHris5aaaa@4CA6@

where *I*(*M*, *q*_*i*_) is the performance of the method *M *for the protein *q*_*i *_(for example, *TFF*(*FUGUE*, *q*_*i*_) is the TFF performance of FUGUE on protein *q*_*i*_).

#### Normalized performance indices

The global performance indices might be affected by the specific make up of the query set, since superfamilies and folds vary greatly in size. In order to reduce a potential bias due to large superfamilies/folds that perform very well, we use also normalized performance indices. To compute these, we divide each index by its upper bound, *i.e. *the total number of proteins in the *SCOP-DB *library that are classified to the same superfamily/fold as the query (except itself). For example, for a query protein *q *that belongs to a fold *F *of size *n*_*F *_the normalized *TFF *measure is given by

*TFF*^*N *^(*M*, *q*) = *TFF *(*M*, *q*)/(*n*_*F *_- 1)

This ratio is essentially the sensitivity of the method *M *on the query *q*, over a match list of size *n*_*F*_. The size of the relevant match list changes with each query.

The resulting ratios are then averaged at the superfamily level so as to obtain a *representative *average performance index per protein superfamily that is bounded between 0 and 1. Finally, the final index is computed by averaging over all the representative indices. I.e. given a query set **Q **= {*q*_1_, ..., *q*_*n*_} that are classified to *k *different superfamilies *F*_1_, ..., *F*_*k *_with *n*_*i *_queries in superfamily *F*_*i*_, then the overall performance of a method *M*, using the normalized index *I*^*N *^is given by

IN(M,Q)=1k∑i=1k1ni∑q∈FiIN(M,q)
 MathType@MTEF@5@5@+=feaafiart1ev1aaatCvAUfKttLearuWrP9MDH5MBPbIqV92AaeXatLxBI9gBaebbnrfifHhDYfgasaacH8akY=wiFfYdH8Gipec8Eeeu0xXdbba9frFj0=OqFfea0dXdd9vqai=hGuQ8kuc9pgc9s8qqaq=dirpe0xb9q8qiLsFr0=vr0=vr0dc8meaabaqaciGacaGaaeqabaqabeGadaaakeaacqWGjbqsdaahaaWcbeqaaiabd6eaobaakiabcIcaOiabd2eanjabcYcaSmXvP5wqSXMqHnxAJn0BKvguHDwzZbqegyvzYrwyUfgaiqqacaWFrbGaeiykaKIaeyypa0ZaaSaaaeaacqaIXaqmaeaacqWGRbWAaaWaaabCaeaadaWcaaqaaiabigdaXaqaaiabd6gaUnaaBaaaleaacqWGPbqAaeqaaaaakmaaqafabaGaemysaK0aaWbaaSqabeaacqWGobGtaaGccqGGOaakcqWGnbqtcqGGSaalcqWGXbqCcqGGPaqkaSqaaiabdghaXjabgIGiolabdAeagnaaBaaameaacqWGPbqAaeqaaaWcbeqdcqGHris5aaWcbaGaemyAaKMaeyypa0JaeGymaedabaGaem4AaSganiabggHiLdaaaa@5BD6@

### Parameter optimization

Our method depends on three parameters: the noise reduction level (the z-score cutoff threshold), the association measure and the distance metric. We consider all possible combinations among five zscore cutoff values (*τ*= 2.5 to 4.5 by increment of 0.5), three association measures that are **P**^*score *^(*zscore*), **P**^*pvalue *^(*pvalue*) and **P**^*prob *^(*prob*), and four distance metrics: *L*_1_, *L*_2_, the JS divergence measure and the new pvalue-distance (PD) measure. Note that the JS measure is only applicable to normalized vectors since it requires the input vector to represent a probability distribution and the PD measure is only applicable to the pvalue vectors.

To find the best parameters we first establish the feature vectors for each sequence in *SCOP-DB*. Each combination of an association function (zscore, pvalue, prob) and a noise threshold *τ*leads to a different set of feature vectors. Next, we compute the distance between the feature vector of each training sequence and the vectors of all sequences in *SCOP-DB*, using one of the distance metrics of section 'Metrics and score functions'. Each combination of feature vectors and a distance metric results in a different set of "match lists". These sets are then evaluated using the performance indices described above. The results are reported in Table 2. For clarity, the best combination of parameters is printed in boldface for each index. To determine the optimal set of parameters, we first examine the effect of the association function. The results are unanimous with all five performance indices: the *pvalue *association measure achieves the best performance, followed by the *prob *association measure. However, the *pvalue *association measure is only effective when coupled with the PD distance metric, while the *prob *association measure seems to produce good results with all distance metrics and especially with *L*_1 _and the JS divergence measure. As for the distance metric, its influence depends on the association function. The PD measure is applicable only to the *pvalue *vectors but it produces excellent results, much better than the traditional *L*_1 _and *L*_2 _metrics. Under the *prob *association measure, *L*_*I *_and *JS *metrics produce the best results while *L*_2 _leads to significantly worse performance. With the two other association measures *L*_1 _and *L*_2 _metrics yield similar results. Finally, the optimal value of *τ*is approximately around 3.5 for most combinations.

**Table 2:** Parameter optimization of the distance-profile method: performance indices based on the training set. The normalized performance indices are given in parentheses and expressed in percentages. For instance, the ROC1-S and TFF indices obtained under the **P**^pvalue ^representation with zscore threshold *τ*= 4 and *L*_2 _distance metric amount to 267 and 363, respectively. The PD measure was evaluated only for *τ*= 3 to 4.5.

**Index**	***τ***	**Association measure/Distance metric**							
		
		*zscore*		*pvalue*			*prob*		
		
		*L*_1_	*L*_2_	*L*_1_	*L*_2_	PD	*L*_1_	*L*_2_	JS
**ROC1-S**	2.5	30 (2.38)	133 (13.87)	10 (0.14)	12 (0.16)		309 (21.67)	75 (1.11)	333 (22.62)
	3	90 (7.95)	156 (16.88)	59 (2.14)	59 (2.14)	453 (41.60)	365 (29.91)	146 (4.75)	375 (30.00)
	3.5	134 (11.98)	164 (18.25)	184 (13.23)	188 (13.55)	**479 (44.63)**	442 (40.04)	262 (16.33)	438 (39.95)
	4	167 (16.82)	164 (18.71)	262 (28.28)	267 (29.00)	475 (44.53)	449 (42.32)	373 (33.58)	438 (42.09)
	4.5	180 (19.75)	159 (18.31)	290 (34.20)	299 (35.35)	467 (42.65)	443 (41.23)	373 (35.86)	427 (41.02)
**ROCI-F**	2.5	31 (2.38)	138 (11.99)	11 (0.16)	13 (0.17)		426 (18.78)	149 (1.31)	460 (20.16)
	3	92 (7.16)	160 (14.43)	59 (2.03)	59 (2.03)	585 (34.69)	478 (26.15)	246 (4.79)	497 (26.30)
	3.5	137 (10.80)	166 (15.23)	185 (12.00)	189 (12.30)	**594 (36.60)**	530 (33.44)	386 (14.49)	536 (33.27)
	4	168 (14.06)	165 (15.39)	264 (23.75)	272 (24.47)	575 (36.40)	477 (34.70)	514 (27.89)	455 (34.04)
	4.5	181 (16.43)	160 (15.24)	295 (28.41)	306 (29.52)	540 (34.97)	461 (33.36)	492 (29.56)	446 (32.89)
**TSS**	2.5	96 (5.38)	183 (17.06)	68 (1.62)	73 (1.78)		433 (29.71)	156 (3.49)	449 (31.46)
	3	144 (9.91)	193 (19.18)	119 (4.10)	119 (4.10)	545 (46.70)	503 (40.85)	209 (7.03)	506 (40.30)
	3.5	174 (13.55)	193 (19.78)	232 (14.84)	234 (15.07)	**546** (47.81)	526 (45.53)	294 (18.12)	531 (45.95)
	4	195 (18.07)	180 (19.46)	306 (31.34)	313 (31.77)	545 **(48.11)**	530 (47.40)	382 (33.80)	530 (47.18)
	4.5	203 (20.74)	172 (18.96)	325 (36.22)	343 (37.64)	509 (45.21)	511 (45.29)	396 (36.67)	509 (45.73)
**TFF**	2.5	171 (5.75)	301 (15.48)	126 (2.00)	136 (2.31)		892 (28.59)	383 (4.41)	915 (30.44)
	3	220 (9.66)	301 (17.02)	150 (4.15)	151 (4.16)	**963 (41.52)**	908 (36.98)	448 (7.52)	913 (36.47)
	3.5	260 (13.22)	302 (17.42)	263 (14.08)	267 (14.32)	875 (40.64)	818 (38.86)	556 (16.84)	831 (39.42)
	4	277 (15.98)	297 (17.40)	350 (26.28)	363 (26.94)	762 (40.05)	751 (39.60)	633 (28.87)	777 (39.38)
	4.5	295 (18.42)	288 (17.14)	401 (30.29)	395 (31.26)	613 (37.55)	616 (37.51)	618 (30.84)	664 (38.03)

In conclusion, these results suggest that the best performance is achieved with the *pvalue *association measure, the pvalue-distance (PD) metric and a zscore cutoff threshold *τ*= 3.5. The same conclusions are reached when using the normalized performance indices. It should be noted that although the *prob *association measure is not as good as the *pvalue *measure (combined with the PD metric), it still produces very good performance overall (with the JS or the *L*_1 _metrics), further justifying the statistical interpretation of our representation and its equivalence with the coefficients of a mixture model over independent sources (as discussed in section 'Conclusions').

**Figure 3:**
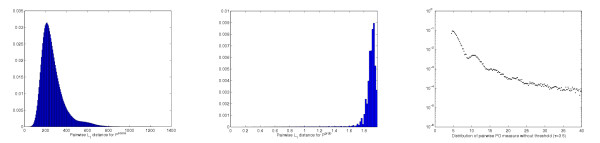
**Left: **Distribution of pairwise distances between feature vectors **P**^*score *^(*τ*= 3.5, *L*_1 _metric). **Middle: **Distribution of pairwise distances between feature vectors **P**^*prob *^(*τ*= 3.5, *L*_1 _metric, distance 2 ignored). **Right: **Distribution of the PD measure for feature vectors **P**^*pvalue *^(*τ*= 3.5). The last distribution is plotted in logscale for distances smaller than 40.

Figure 3a,b illustrate the distribution of the pairwise *L*_1_-distances between proteins under the representations **P**^*score *^and **P**^*prob*^, respectively. We observe that the pairwise distance between feature vectors **P**^*score *^spreads over a very large range and it is difficult to set a natural threshold below which feature vectors can be considered similar. In contrast, for **P**^*prob*^, about 95% of the pairwise distances are equal to 2, the maximum *L*_1 _distance. This is the distance between pairs of normalized feature vectors (probability distributions) whose set of non-zero features do not overlap. The distribution shown in Figure 3b only focuses on those pairwise distances smaller than 2. The combination of noise reduction, pvalue conversion and normalization procedures effectively delimits the range of the pairwise distances, and any distance smaller than 2 indicates common features between the feature vectors.

Figure 3c shows the empirical distribution of the PD measure between feature vectors **P**^*pvalue*^. The distribution is multi-modal (see Appendix for a more detailed discussion). This emirical distribution is used to estimate the significance of the PD measure.

### Performance Comparison

We compare the performance of our algorithm against several existing algorithms, namely FUGUE [30], BLAST [42] and PSIBLAST [5]. Table 3 reports the results for all algorithms based on the training sequences. The DP algorithm was run with the optimal parameters that were determined in the previous section. With BLAST, we simply compare the query sequence with all sequences in *SCOP-DB*. With FUGUE, we thread the query sequence into each one of the structural templates of the proteins in *SCOP-DB*. For reference, we also list the results obtained with the structure comparison algorithm URMS [43]. The URMS results provide a rough upper bound on the expected performance since the algorithm is directly using the structural information which underlies many of the homology relationships in SCOP.

**Table 3:** Comparison between BLAST, PSIBLAST, FUGUE, *DP-FUGUE *(*τ*= 3.5, PD, *pvalue*) and URMS on the training set. The normalized indices (in percentages) are given in parentheses. PSIBLAST's parameters were set to *h *= 1*e*^-5^, *e *= 100 and *j *= 10 (although no improvement was observed after the fourth iteration). FUGUE was run using the default parameters.

**Index**	**BLAST**	**PSIBLAST (2 to 4 iterations)**			**FUGUE**	***DP-FUGUE***	**URMS**
		**2 iterations**	**3 iterations**	**4 iterations**			

ROC1-S	212 (25.64)	265 (29.80)	281 (30.91)	279 (30.68)	296 (35.03)	479 (44.63)	610 (60.20)
ROC1-F	212 (21.65)	265 (24.58)	281 (25.23)	279 (25.12)	306 (28.64)	594 (36.60)	719 (51.58)
TSS	278 (29.32)	315 (33.48)	335 (34.54)	335 (34.54)	422 (40.20)	546 (47.81)	797 (69.07)
TFF	344 (24.76)	386 (28.08)	410 (28.84)	409 (28.81)	659 (34.72)	875 (40.64)	1681 (67.30)

As Table 3 shows, FUGUE improves the ROC1-S and ROC1-F indices by 6 and 9% over PSIBLAST while the TSS and TFF indices are increased by a magnitude of 24 to 60%. The distance-profile method *DP-FUGUE *improves over PSIBLAST by more than 60% on all indices. With respect to FUGUE, it increases by 62 and 94% the ROC1-S, ROC1-F indices and about 30% for both the TSS and TFF indices. This margin of improvement over FUGUE is also maintained over the test set (Table 4). Quantitatively, the ROC1-S and ROC1-F are improved by about 90% and the TSS, TFF indices by about 42%. This is a substantial improvement that is larger than the relative improvement of PSIBLAST with respect to BLAST or that of FUGUE with respect to PSIBLAST, thus indicating that the statistical fingerprints of the distance-profile representation encode more information and are more sensitive than a direct comparison of the objects they operate on.

**Table 4:** Comparison between BLAST, PSIBLAST, FUGUE and *DP-FUGUE *(*τ*= 3.5, PD, *pvalue*) on the test set. The normalized indices (in percentages) are given in parentheses.

**Index**	**BLAST**	**PSIBLAST**	**FUGUE**	***DP-FUGUE***
ROC1-S	6658 (24.42)	9266 (28.75)	12143 (35.10)	23307 (45.55)
ROC1-F	6694 (18.86)	9319 (21.93)	12620 (26.91)	24693 (35.21)
TSS	11379 (28.51)	15149 (32.62)	21176 (41.03)	30238 (49.62)
TFF	13986 (22.72)	18172 (25.85)	28473 (33.68)	40376 (40.66)

**Figure 4:**
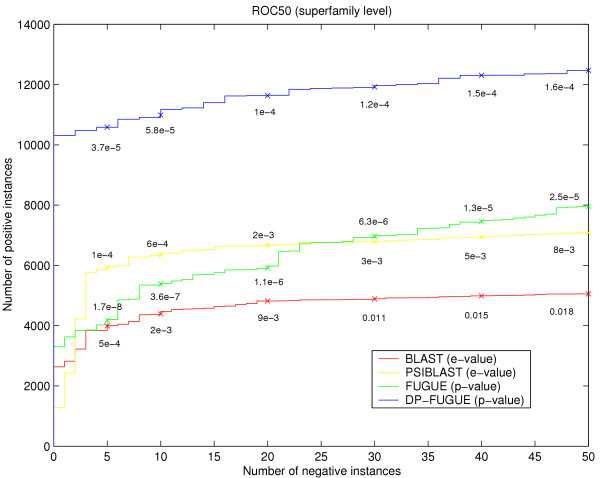
**ROC50 curves over the test set**. Along each curve we indicate the e-value (or z-score) at different rates of false positives. Thus, at each significance threshold one can estimate the ratio between the number of true and false matches.

Figure 4 displays the ROC50 curve for each algorithm. As opposed to the previous indices that are computed per query and then averaged, these ROC curves are generated by aggregating all pairwise similarities computed with a given method and sorting the list in descending order of significance. Each curve plots the cumulative number of positives versus the cumulative number of negatives until 50 negatives are observed (where positives are set at the superfamily level). The results agree with the previous indices, and the distance-profile method significantly outperforms all other methods as the amount of positives detected reaches almost 12,500 (compared to 8,000 with FUGUE and 7,000 with PSIBLAST) when the number of negatives reaches 50. A similar ROC50 curve is observed at the fold level as well. The results above demonstrate that our method can effectively detect remote homologies. However, an interesting question that one might raise is: under what scenarios the distance-profile representation can improve the results over the original method from which it is derived. To answer this question, we contrast the performance of *DP-FUGUE *and FUGUE for individual queries. Specifically, for each query in the test set we plot the normalized TSS index of *DP-FUGUE *vs. the TSS index of FUGUE for the same query. As the graph shows, in most cases the improvement occurs when the TSS index of the original method is above 20%, and on average one may expect an improvement if it is above 10% (i.e. if as little as 10% of the superfamily are observed in the proximity of the query within a neighborhood as big as the superfamily).

### The distance-profile representation over sequence-profile metrics

To test the effectiveness of the distance-profile method on other types of input we applied it to feature vectors that were generated with PSIBLAST, a sequence to profile alignment algorithm [5]. The feature values are set to the *log *(*evalue*) of the similarity score, as reported by PSIBLAST after four iterations, unless the program converged before (as Table 3 demonstrates, the performance plateaus after four iterations). The PSIBLAST evalue-based feature vectors are processed in a similar fashion to the FUGUE zscore-based feature vectors. Each evalue *e *is mapped to its corresponding pvalue *pvalue*(*e*) = 1 - *exp*(-*e*) as in [44] and the value of the corresponding feature is defined as 1 - *pvalue*(*e*) = *exp*(-*e*). If the evalue is greater than a given threshold *τ*' then we reset the value of the feature. Finally, the normalization converts the resulting feature vectors to probability distributions.

The parameters are optimized using a similar procedure to the one described in section 'Parameter optimization'. The optimal parameters are sought among the combinations of 4 evalue cutoff thresholds (*τ*' = 0.01, 0.1, 1, 10), two association measures (**P**^*pvalue *^(*pvalue*), **P**^*prob *^(*prob*)) and three possible metrics (*L*_1_, *L*_2 _and JS divergence measure). In this case, the best combination based on the training set is (*τ*' = 10, *L*_1_, *prob*).

Table 5 compares the performance indices associated to PSIBLAST after 4 iterations with *DP-PSIBLAST *with parameters (*τ*' = 10, *L*_1_, *prob*). The distance-profile representation clearly improves the performance compared to PSIBLAST (increases the indices by about 20% to 38%) and even to FUGUE in some cases, but is not as powerful as the distance-profile representation when applied to FUGUE (compare to the results of Table 3). It is also interesting to note that the overall improvement of *DP-PSIBLAST *over PSIBLAST seems smaller than that of *DP-FUGUE *over FUGUE. As we showed at the end of the previous section, the magnitude of improvement depends on the initial success of the association measure among the queries and the reference set. Since PSIBLAST yields less instances with performance that exceeds the minimal threshold (i.e. cases that can be improved using the DP representation), it follows that the overall improvement of DP representation on PSIBLAST is less significant compared to that over FUGUE. Detailed examples are discussed in the Appendix.

**Table 5:** Comparison between PSIBLAST_4 _(PSIBLAST with 4 iterations) and *DP-PSIBLAST *(*τ*' = 10, *L*_1_, *prob*) based on the training set. The normalized indices (in percentages) are given in parentheses.

**Index**	**PSIBLAST_4_**	***DP-PSIBLAST***
ROC1-S	279 (30.68)	339 (35.16)
ROC1-F	279 (25.12)	339 (28.86)
TSS	335 (34.54)	462 (39.63)
TFF	409 (28.81)	510 (33.06)

### The effect of multiple sequence alignment on the performance

All our experiments were performed in a single-query mode. I.e. in each test case a single query sequence is compared against the database. However, there are multiple reports [40,45,46] that suggest that significant performance gain can be obtained when using a multiple sequence alignment (MSA) or a sequence profile as a query.

To test the effect of MSA on the performance we had the change our experimental setup. We tested the impact of the new setting on PSIBLAST and FUGUE based on 7 SCOP families that were randomly chosen from all families for which the performance of PSIBLAST and FUGUE was poor. For each family we generated a MSA of all sequences in the family using CLUSTALW [47]. Each MSA was also converted to a position specific scoring matrix (profile). These MSA were used as queries for FUGUE (instead of the individual sequences) and the profiles as input for PSIBLAST, in search for related sequences in *SCOP-DB*. To fairly compare the results of PSIBLAST and FUGUE in the MSA mode to the DP method we had to run our method under a similar setup. The "MSA" mode of the DP method utilizes the information from all the sequences in a protein family in the same spirit a MSA does so, by combining the distance profiles associated to each member of the SCOP family. For each sequence in *SCOP-DB*, we take the average of its distance versus each member of the family in question in order to compute the "family-specific" distances with respect to *SCOP-DB*.

In Table 6, we summarize for each of the SCOP families the performance of FUGUE, PSIBLAST and *DP-FUGUE *under the MSA-query mode. Family members are not counted since they were already used to build the MSA. The adjusted indices thus indicate how many *remote *homologs are discovered in the MSA-query mode. For comparison we also report the average performance under the single-query mode. As the results demonstrate, the MSA mode improves over the standard single mode, and in most cases our method performs better than FUGUE and PSIBLAST, in particular in terms of the TSS index (reflecting how well remote homologs are clustered at the top of the ranked list). It should be noted that when we analyzed FUGUE in a similar manner to *DP-FUGUE *(i.e. by averaging over the individual family members) the results improved over the MSA mode of FUGUE, but not as much as *DP-FUGUE *in MSA mode.

**Table 6:** Performance indices of FUGUE, PSIBLAST and *DP-FUGUE *under the single and the MSA query modes. The counts exclude those sequences in the SCOP family in question (that were used to build the MSA). In single query mode we report the average performance. Results are reported using the ROC1-S and the TSS indices. Similar trends were observed with the ROC1-F and the TFF indices.

**Family**	**Mode**	**ROC1-S**			**TSS**		
		
		**FUGUE**	**PSIBLAST**	***DP-FUGUE***	**FUGUE**	**PSIBLAST**	***DP-FUGUE***
a.3.1.1	single	0.4	0.4	2.6	2.8	1.7	3.5
	MSA	0	2	4	2	3	4
a.3.1.4	single	2.3	1.6	11	6.3	5.3	12.3
	MSA	1	4	12	4	7	12
a.39.1.5	single	2.8	5	12	7.7	9.5	13.4
	MSA	15	5	14	16	9	15
b.47.1.4	single	1.6	0	22.3	8.6	1.6	22.3
	MSA	3	1	23	17	5	23
c.2.1.3	single	0.2	0.1	3.1	7.6	5.4	15.4
	MSA	2	0	0	14	6	26
c.3.1.2	single	1.8	0.7	9	8.7	5.5	14.1
	MSA	1	3	12	20	8	19
c.47.1.2	single	4.1	2.3	16.1	8.8	4.8	19.3
	MSA	12	5	24	19	7	25

We should comment that the MSA setup differs from our original idea of using the DP representation to perform *unsupervised clustering *of objects based on their distance profile. In the unsupervised learning mode we do not have information on the family association of each sequence, and therefore it is difficult to define the exact set of related sequences from which to generate a multiple sequence alignment.

### Superfamily and fold prediction with the distance-profile method

We tested the power of our method on a new set of protein sequences that were added to SCOP after we compiled our benchmark. Our goal was to test if the method can classify new sequences to their correct class. The new set consists of proteins in release 1.67 of SCOP that were not included in our *SCOP-DB *dataset (based on release 1.57 of SCOP) and either belong to *new *families within *existing *superfamilies, *new *superfamilies within *known *folds or completely *new *folds. For instance, the family b.2.3.4 did not exist in the SCOP 1.57 database. This family is part of the b.2.3 superfamily that in release 1.57 contains the families b.2.3.1, b.2.3.2, b.2.3.3 and a total of 5 representatives in our reference set.

In total we found 624 sequences belonging to 453 new families within known superfamilies, 267 sequences associated to 182 new superfamilies within known folds and 375 sequences in 245 new folds, all with less than 40% identity between pairs of sequences. Each one of these new sequences was compared against all the sequences in *SCOP-DB *using all of the methods evaluated in this paper, and the matches were sorted based on the score or distance, as before. To apply the distance-profile method the sequences were first processed and mapped to feature vectors as described in section 'Results'.

Table 7 summarizes the results using our four performance indices. We note that only the ROC1-F and TFF indices are reported for sequences belonging to new superfamilies within known folds (since none of these new sequences have superfamily members in the reference set). As the table demonstrates, for sequences belonging to new families, our method consistently improves homology detection. The improvement over FUGUE is significant with the ROC1-S and ROC1-F indices increasing by more than 100% (40% with normalized measures) while the TSS and TFF indices are improved by more than 30% (15% in normalized form). For sequences belonging to new superfamilies *DP-FUGUE also *improves over the three other methods, however, the improvement is smaller. As for the sequences in new folds, our experiment indicates that in most cases *DP-FUGUE *cannot improve the results, the reason being that FUGUE itself typically is unable to detect any significant match with members of our reference set. Finally, we conducted another experiment to study the performance of our method in detecting distant homologs that share little sequence identity. In order to do so, we selected from the new SCOP sequences those having less than 20% sequence identity with respect to the proteins in our reference set. We obtained 277 such sequences associated to new families and 103 to new superfamilies. Table 8 again shows that our method outperforms BLAST, PSIBLAST and FUGUE, with the exception of the TFF index over sequences that belong to new superfamilies within known folds, where a slight decrease is observed. This can be explained by the fact that FUGUE itself does not perform satisfactorily (the normalized TFF is only about 2.4%).

**Table 7:** Class prediction for new SCOP sequences. Comparison between BLAST, PSIBLAST, FUGUE, *DP-FUGUE *(*τ*= 3.5, PD, *pvalue*). The normalized indices (%) are given in parentheses.

	**Index**	**BLAST**	**PSIBLAST**	**FUGUE**	***DP-FUGUE***
New families	ROC1-S	187 (7.03)	211 (7.71)	380 (16.35)	847 (22.56)
	ROC1-F	216 (5.68)	239 (6.34)	450 (11.65)	1185 (17.98)
	TSS	419 (8.97)	458 (10.31)	1014 (24.66)	1412 (27.58)
	TFF	715 (7.78)	827 (8.79)	2518 (19.61)	3512 (22.69)

New superfamilies	ROC1-F	8 (0.51)	11 (0.56)	42 (0.89)	187 (1.24)
	TFF	156 (1.19)	169 (1.25)	853 (3.58)	1097 (3.33)

**Table 8:** Class prediction for new SCOP sequences with little sequence identity. In this case the analysis is limited to new SCOP sequences with less than 20% sequence identity with respect to the reference set.

	**Index**	**BLAST**	**PSIBLAST**	**FUGUE**	***DP-FUGUE***
New families	ROC1-S	41 (2.52)	43 (3.14)	73 (8.44)	156 (11.77)
	ROC1-F	43 (1.95)	45 (2.57)	75 (6.41)	181 (9.89)
	TSS	94 (4.85)	101 (5.36)	208 (15.80)	255 (17.11)
	TFF	142 (3.90)	161 (4.49)	364 (12.42)	602 (13.77)

New Superfamilies	ROC1-F	1(0.09)	1 (0.09)	6 (0.36)	50 (0.738)
	TFF	20 (0.80)	23 (0.92)	119 (2.39)	108 (2.22)

## Conclusion

We study a new method for remote homology detection that utilizes global information on the proximity of entities in the protein space. Our method relies on the distance-profile representation of proteins and protein families that maps each query protein to a high-dimensional feature space, where the coordinates are determined by some association measures with respect to a reference set. These vectors are then processed and transformed to sparse feature vectors that are treated as statistical fingerprints of the query proteins. We experimented with several different types of association measures and demonstrated how an adequate choice of distance metric combined with a proper transformation of the feature vectors through noise reduction, pvalue conversion and normalization can greatly increase the performance of homology recognition (or prediction) compared to the existing approaches.

Interestingly, excellent performance is obtained with normalized feature vectors that correspond to probability distributions. The success of the distance-profile method in general and especially when using probability distributions suggests a relation to mixture models [48]. Specifically, one can consider this representation as the coefficients of a mixture model or of a functional expansion, similar to the Taylor polynomial expansion. Given a set of basis functions such as polynomial functions one can span the complete space of continuous well-behaved functions with the right coefficients. The same principle applies here as our reference set essentially defines a set of basis functions. In statistical terms, each element of the reference set induces a different probability distribution over the protein sequence space. In our experiments the reference set is composed of protein structures, each one can be perceived as a different generative model. The likelihood of generating a sequence according to a model can be estimated by computing the probability that the sequence will fold into the corresponding structure, as measured with the pvalue association measure over the threading similarity score. Although these probability distributions (that correspond to different elements in the reference set) do not necessarily meet the requirement of orthogonality to be considered "basis functions", a sufficiently diverged set of proteins is expected to have the desired properties. It has yet to be defined more precisely what sufficiently diverged means and the minimal required diversity.

One intriguing aspect that has not been fully addressed is the interaction between the association function, the distance metric and the zscore cutoff value. In some cases the coupling is not surprising. For example, when the *prob *association function is used, the *L*_2 _metric clearly underperforms compared to *L*_1 _and *JS *metrics, as expected, since the vectors compared correspond to probability distributions. With the other association measures, *L*_1 _and *L*_2 _perform similarly. Study of the theoretical aspect of this phenomenon will help understand how to take further advantage of these feature vectors to cluster the protein sequences more accurately.

A word of caution is in order here regarding the evaluation. While SCOP is considered the gold standard, it is not perfect and often one can find mis-classifications [7,24,49]. While it is hard to estimate the exact rate of errors, it is unlikely that they exceed thousands and we do not anticipate the results to change drastically even if these mis-classification were corrected.

We should also mention that the DP representation is not effective for detailed, atom-resolution prediction of 3D structure or for site-specific functional annotation, since it cannot produce alignments. Another weakness is that the distance-profile representation and the new pvalue-distance measure may fail to distinguish two proteins if they have almost identical "preferences" for the known structures but different preferences for other, unknown structures that are yet to be determined. However, given the current size of the protein structure space, it is expected that for most proteins the available structural information (as embodied in our reference set) is sufficient to estimate their proximity. Indeed, only 20% of the new SCOP 1.67 sequences were assigned to new folds, Clearly, as more structures are determined and integrated into the reference set, the distance-profile representation is expected to improve.

One potential contribution of this work is the possibility of combining the transformations described in section 'Processing feature vectors' to the feature vectors used by existing kernel methods. These techniques can effectively reduce noise and increase the accuracy of classification of the feature space. In addition, since the feature vectors are typically sparse after noise reduction, an efficient computation of the kernel function can be implemented in a high-dimensional feature space.

Finally, a major advantage of the distance-profile representation is in its great flexibility. The underlying association measure can be based on sequence, structure, predicted function, threading, or any other similarity measure. Clearly, more distinctive association measures will create better representations. Indeed, the FUGUE zscore is clearly a better choice than the PSIBLAST evalue since FUGUE exploits sequence-structure alignment information rather than just sequence alignment information. If the association measure can report a significance value (such as zscore or evalue) that emphasizes extremes and pinpoint the interesting cases, the statistical measure will be preferred over the raw score. This is especially useful when the raw score is meaningless by itself. In these cases one needs a yardstick or a scale to tell what is close and what is far and the statistical estimates provide such a scale. Nevertheless, it is important to note that the distance-profile representation and the induced similarity measure are quite robust and work well even with raw association scores, noisy or corrupted data, and weak signal-to-noise ratio [25]. All our data, including the FUGUE results, the PSIBLAST results and the feature vectors are available at [50].

## Authors' contributions

CK implemented the model, ran experiments, compared to other models, analyzed the result sets and wrote parts of the manuscript. GY conceived of the study, designed the model and the experiments, analyzed results and drafted the manuscript.

## Appendix

### The sequence-structure association measure

Historically, sequence-structure threading [4] was proposed as an alternative approach to predict the structural fold of polypeptide chains. In contrast to *ab initio *strategies that exploit secondary structure prediction, energy minimization and molecular dynamics to predict the structure of a protein sequence, sequence-structure threading consists of finding native-like folding structure(s) for a query protein from a database of *known *structures. Its motivation originates from the observation that proteins adopt a limited number of spatial architectures and that larger proteins are frequently composed of modules that can be found in other proteins.

To perform sequence-structure threading of a query sequence, the process typically starts by obtaining a set of structural conformations from a database of known structures. The amino acid sequence of the query protein is aligned to each conformation in search of an alignment that would produce the minimal total energy (that depends on the structural environment of each reside, or the types of neighboring residues as determined by the sequence-structure alignment). The most likely candidate conformations are the ones yielding the lowest energy. If the energy values are significantly low compared for example to those obtained for shuffled sequences, then these structural conformations can be considered as compatible with the query sequence.

Many different approaches and implementations of threading algorithms have been proposed in the past [30,51-54]. The exact details of the alignment algorithm and the computation of the total energy vary from one method to another. Unfortunately, most of them are not publicly available to allow an extensive comparison of sequences and structures. Of the few that are available, we chose FUGUE for our study. FUGUE [30] is a sequence-structure alignment algorithm that uses environment-specific substitution tables and structure-dependent gap penalties to evaluate the alignment score. It switches between local and global alignment based on the ratio between the length of the query sequence and that of the structural profile. Previous experimental results have shown that FUGUE outperforms other methods in fold recognition such as PSIBLAST [5], SAM-PSIBLAST [32], HMMER-PSIBLAST [33], THREADER [51] and GenTHREADER [53]. It is worth mentioning that FUGUE should be considered as a structure-based sequence alignment algorithm rather than a sequence-structure alignment algorithm *per se*, since it does not involve the definition and the minimization of any energy function to measure the goodness of fit of the query sequence to the template structure. However, its use of substitution tables and structure-dependent parameters provides additional information which is unavailable to pure sequence-based methods. In FUGUE, the compatibility of each sequence-structure pair is assessed through the *zscore*, which measures the departure of the observed threading score value from its mean, normalized by the standard deviation (where the mean and standard deviation are computed based on the distribution of alignment scores over shuffled sequences. Zscores of meaningful matches are then shifted such that the minimal zscore starts at 0. It is claimed that a zscore less than 2 typically implies an uncertain match between the template structure and the query amino acid sequence, and a zscore larger than six implies an almost certain match between the protein and the template folding structure [30].

It should be noted that threading measures are asymmetric. Given two proteins, *A *and *B *with known structures, then *S*(*B*, *A*) does not necessarily equal to *S*(*A*, *B*). Actually, for most protein pairs the equality *S*(*B*, *A*) = *S*(*A*, *B*) does not hold, as different protein structures have different "sequence capacities". These capacities affect the ability of an arbitrary sequence to conform with the given structure, and therefore also the probability that the structure will be energetically favorable for the given sequence. However, the asymmetry may be a fundamental feature of the protein space that we may want to preserve and study later on in our analysis.

### The statistical significance of the PD measure

**Figure 6:**
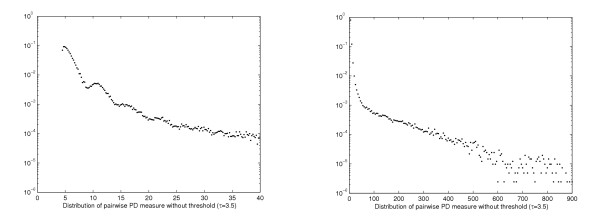
**Distribution of pvalue-distances between feature vectors P^*pvalue *^with thresholding (*τ*= 3.5)**. Thresholding introduces complex effect on the distribution of the PD measure and makes the derivation of its significance level difficult. **Right: **The complete distribution is plotted in logscale. The linear correlation in logscale suggests an exponential decay. **Left: **Zoom-in on the range [0,40]. The distribution is multi-modal due to the contributions of a different number of elements to sum as in Equation (4). The smallest nonzero PD measures start at approximately 4.426, which corresponds to a match of two feature vectors **P**^*pvalue *^at one entry where the z-score is equal to 3.5, *i.e. *a pvalue of 0.0120 or -log *pvalue *= 4.426. The distribution decreases rapidly and increases again at PD ≈ 9, corresponding to a match of two feature vectors at two distinct entries with zscore ≈ 3.5. The pattern repeats periodically as the number of significant entries common to both feature vectors increases.

We computed the distribution of pvalue-distances (PD) between pairs of feature vectors **P**^*pvalue *^of unrelated protein sequences. The distribution is a complex one, and features multiple modes (Figure 6a). Each mode corresponds to the occurrence of one additional term in the summation contributing to the final value of the PD measure as described in Eq (4), *i.e. *the two feature vectors share one additional entry where the pvalues are both significant. One may characterize the PD measure as the sum of a random number of EVD random variables. Unfortunately, analysis of such a distribution is generally intractable and therefore we can only estimate the significance level of our PD measure based on the empirical distribution.

In Figure 6b, we plot the distribution of the PD measure over its full range (up to 900) in log scale. We observe that the frequency of occurrence of large PD values (typically above 100) decreases exponentially, *i.e. *linearly in log scale (the increasingly dispersed pattern at large PD value > 600 is mainly due to data sparsity). This phenomenon can be explained by examining again the definition of the PD measure given in (4). If we consider a zscore as a random variable, its associated pvalue is a random variable uniformly distributed between 0 and 1. Based on the assumption that pi1
 MathType@MTEF@5@5@+=feaafiart1ev1aaatCvAUfKttLearuWrP9MDH5MBPbIqV92AaeXatLxBI9gBaebbnrfifHhDYfgasaacH8akY=wiFfYdH8Gipec8Eeeu0xXdbba9frFj0=OqFfea0dXdd9vqai=hGuQ8kuc9pgc9s8qqaq=dirpe0xb9q8qiLsFr0=vr0=vr0dc8meaabaqaciGacaGaaeqabaqabeGadaaakeaacqWGWbaCdaqhaaWcbaGaemyAaKgabaGaeGymaedaaaaa@308F@ and pi2
 MathType@MTEF@5@5@+=feaafiart1ev1aaatCvAUfKttLearuWrP9MDH5MBPbIqV92AaeXatLxBI9gBaebbnrfifHhDYfgasaacH8akY=wiFfYdH8Gipec8Eeeu0xXdbba9frFj0=OqFfea0dXdd9vqai=hGuQ8kuc9pgc9s8qqaq=dirpe0xb9q8qiLsFr0=vr0=vr0dc8meaabaqaciGacaGaaeqabaqabeGadaaakeaacqWGWbaCdaqhaaWcbaGaemyAaKgabaGaeGOmaidaaaaa@3091@ are independent (which is true if the two feature vectors are randomly drawn from our database), max(pi1
 MathType@MTEF@5@5@+=feaafiart1ev1aaatCvAUfKttLearuWrP9MDH5MBPbIqV92AaeXatLxBI9gBaebbnrfifHhDYfgasaacH8akY=wiFfYdH8Gipec8Eeeu0xXdbba9frFj0=OqFfea0dXdd9vqai=hGuQ8kuc9pgc9s8qqaq=dirpe0xb9q8qiLsFr0=vr0=vr0dc8meaabaqaciGacaGaaeqabaqabeGadaaakeaacqWGWbaCdaqhaaWcbaGaemyAaKgabaGaeGymaedaaaaa@308F@, pi2
 MathType@MTEF@5@5@+=feaafiart1ev1aaatCvAUfKttLearuWrP9MDH5MBPbIqV92AaeXatLxBI9gBaebbnrfifHhDYfgasaacH8akY=wiFfYdH8Gipec8Eeeu0xXdbba9frFj0=OqFfea0dXdd9vqai=hGuQ8kuc9pgc9s8qqaq=dirpe0xb9q8qiLsFr0=vr0=vr0dc8meaabaqaciGacaGaaeqabaqabeGadaaakeaacqWGWbaCdaqhaaWcbaGaemyAaKgabaGaeGOmaidaaaaa@3091@) follows a triangular distribution and -log max(pi1
 MathType@MTEF@5@5@+=feaafiart1ev1aaatCvAUfKttLearuWrP9MDH5MBPbIqV92AaeXatLxBI9gBaebbnrfifHhDYfgasaacH8akY=wiFfYdH8Gipec8Eeeu0xXdbba9frFj0=OqFfea0dXdd9vqai=hGuQ8kuc9pgc9s8qqaq=dirpe0xb9q8qiLsFr0=vr0=vr0dc8meaabaqaciGacaGaaeqabaqabeGadaaakeaacqWGWbaCdaqhaaWcbaGaemyAaKgabaGaeGymaedaaaaa@308F@, pi2
 MathType@MTEF@5@5@+=feaafiart1ev1aaatCvAUfKttLearuWrP9MDH5MBPbIqV92AaeXatLxBI9gBaebbnrfifHhDYfgasaacH8akY=wiFfYdH8Gipec8Eeeu0xXdbba9frFj0=OqFfea0dXdd9vqai=hGuQ8kuc9pgc9s8qqaq=dirpe0xb9q8qiLsFr0=vr0=vr0dc8meaabaqaciGacaGaaeqabaqabeGadaaakeaacqWGWbaCdaqhaaWcbaGaemyAaKgabaGaeGOmaidaaaaa@3091@) is an exponential random variable. Therefore, -log *PD *can be viewed as a sum of exponential random variables. From the statistical literature, we know that the sum of *k *independent and identically distributed exponential random variables with parameter *α *can be modeled by the Gamma function.

fG(x;k,α)=xk−1e−x/ααkΓ(k)     (5)
 MathType@MTEF@5@5@+=feaafiart1ev1aaatCvAUfKttLearuWrP9MDH5MBPbIqV92AaeXatLxBI9gBaebbnrfifHhDYfgasaacH8akY=wiFfYdH8Gipec8Eeeu0xXdbba9frFj0=OqFfea0dXdd9vqai=hGuQ8kuc9pgc9s8qqaq=dirpe0xb9q8qiLsFr0=vr0=vr0dc8meaabaqaciGacaGaaeqabaqabeGadaaakeaacqWGMbGzdaWgaaWcbaGaem4raCeabeaakiabcIcaOiabdIha4jabcUda7iabdUgaRjabcYcaSiabeg7aHjabcMcaPiabg2da9maalaaabaGaemiEaG3aaWbaaSqabeaacqWGRbWAcqGHsislcqaIXaqmaaGccqWGLbqzdaahaaWcbeqaaiabgkHiTiabdIha4jabc+caViabeg7aHbaaaOqaaiabeg7aHnaaCaaaleqabaGaem4AaSgaaOGaeu4KdCKaeiikaGIaem4AaSMaeiykaKcaaiaaxMaacaWLjaWaaeWaaeaacqaI1aqnaiaawIcacaGLPaaaaaa@4F41@

For large *x*, we have

log⁡fG(x;k,α)=ε−xα+(k−1)log⁡x∝−xα     (6)
 MathType@MTEF@5@5@+=feaafiart1ev1aaatCvAUfKttLearuWrP9MDH5MBPbIqV92AaeXatLxBI9gBaebbnrfifHhDYfgasaacH8akY=wiFfYdH8Gipec8Eeeu0xXdbba9frFj0=OqFfea0dXdd9vqai=hGuQ8kuc9pgc9s8qqaq=dirpe0xb9q8qiLsFr0=vr0=vr0dc8meaabaqaciGacaGaaeqabaqabeGadaaakeaacyGGSbaBcqGGVbWBcqGGNbWzcqWGMbGzdaWgaaWcbaGaem4raCeabeaakiabcIcaOiabdIha4jabcUda7iabdUgaRjabcYcaSiabeg7aHjabcMcaPiabg2da9iabew7aLjabgkHiTmaalaaabaGaemiEaGhabaGaeqySdegaaiabgUcaRiabcIcaOiabdUgaRjabgkHiTiabigdaXiabcMcaPiGbcYgaSjabc+gaVjabcEgaNjabdIha4jabg2Hi1kabgkHiTmaalaaabaGaemiEaGhabaGaeqySdegaaiaaxMaacaWLjaWaaeWaaeaacqaI2aGnaiaawIcacaGLPaaaaaa@56FA@

where *ε *is an additive constant. Hence, log *f*_*G*_(*x*; *k*, *α*) approximately decreases with *x *in a linear fashion. In our case, clearly the summands in (4) are not independent of each other since the reference set is composed of groups of related sequences. Nonetheless, one may expect that the dependency is not too strong because these sequences share less than 40% sequence identity. The plot indeed suggests that the distribution of the PD measure follows this trend.

### Examples of homology detection

Table 9 reports the results for four specific queries. For simplicity, proteins are designated by their SCOP family name followed by a number indicating their relative position in the original SCOP file. For instance, the first protein in the SCOP file belonging to the family a.1.1.1 is denoted by a.1.1.1.1, and so on. The queries were picked so as to demonstrate that the best performance for a given combination of a query protein and a performance index can be obtained with any of the methods we tested. However, on average the distance-profile method is significantly more sensitive and cases like c.37.1.3.6 are rare.

**Table 9:** Homology detection for a few example query proteins. For each query and method we report the results using the performance indices described in the 'Discussion' section.

**Protein**	**Index**	**BLAST**	**PSIBLAST**	**FUGUE**	***DP-PSIBLAST***	***DP-FUGUE***
*b.1.1.1.14*	ROC1-S	14	26	30	**78**	66
	ROC1-F	14	26	30	**78**	66
	TSS	31	54	64	86	**99**
	TFF	39	62	78	90	**127**
*g.3.11.1.8*	ROC1-S	4	4	7	6	**10**
	ROC1-F	4	4	7	6	**10**
	TSS	5	5	11	6	**20**
	TFF	7	7	**24**	8	**24**
*c.2.1.2.26*	ROC1-S	2	2	5	2	**28**
	ROC1-F	2	2	5	2	**28**
	TSS	6	6	25	28	**42**
	TFF	6	6	25	28	**42**
*c.37.1.13.6*	ROC1-S	3	**4**	3	**4**	3
	ROC1-F	3	**4**	3	**4**	3
	TSS	7	7	**9**	7	4
	TFF	7	7	**9**	7	4

**Figure 5:**
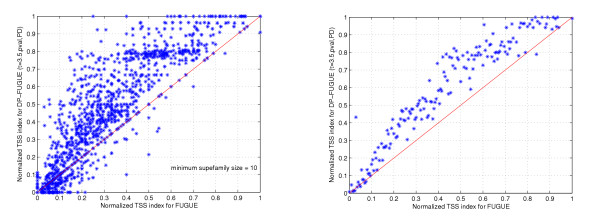
**Comparison of the normalized TSS index of FUGUE and *DP-FUGUE***. **Left: **Scatter plot based on all queries in the test set that belong to SCOP superfamilies containing at least 10 members. **Right: **Averaged scatter plot (for each TSS value of FUGUE we average all TSS indices of *DP-FUGUE *that are associated with that value). Points situated above the diagonal represent successful cases where the TSS index is improved when using the DP representation and vice versa.

Tables 10 and 11 list the closest neighbors of the protein queries *d*2*tgf__ *(*Transforming growth factor alpha *(designated g.3.11.1.8) and *d*1*hdoa_ *(*Biliverdin IX beta reductase*) (designated c.2.1.2.26) with each one of the five competing methods. As these demonstrate, a significantly larger number of proteins that are biologically related to the query sequence are placed at the top of the neighbor list with the distance-profile method. For example, the protein domain g.3.11.1.8 belongs to the superfamily g.3.11 which contains 26 entries in the *SCOP-DB *database. Both PSIBLAST and BLAST detect 4 true positives (TP) before encountering the first false positive (FP) d.158.1.1.1 (SCOP domain *d*1*a6q__*), whereas *DP-PSIBLAST *extends the ROC1-S index to 6. FUGUE starts with 7 TP at the top of the sorted list and our *DP-FUGUE *method further improves the index to 10 TP. The differences for the protein domain c.2.1.2.26 are even more substantial, with BLAST, PSIBLAST and *DP-PSIBLAST *reporting only two TP before the first FP, FUGUE reporting 5 TP, while *DP-FUGUE *reporting 28 TP, thus significantly enhancing the clustering of the members of the superfamily c.2.1. We observe that the performance of the DP method depends on the configuration of the sorted list obtained using the initial association measure. Our method works best when some true positives can be found among the top ones on the list, even if proceeded by or mixed with false positives; it refines the results by clustering even closer the similar elements while pushing further away the false positives. Our method does not improve the results in the case of c.37.1.13.6. However, we note that this is a difficult case of homology detection since there are more than 86 members in the superfamily c.37.1 while all methods detect only a very few true positives at the top of the sorted list. There are several cases where FUGUE lists a FP at the top of the list but a couple of TPs are among the closest ones. In these cases, DP can enhance the mapping and improve over FUGUE, as is also demonstarted in Figure 5. However, when there is only one TP at the top of the list, our method does not always improve over FUGUE. This is most difficult when the top true positive match is only marginally higher than the first false positive.

**Table 10:** Closest neighbors of *d*2*tgf __ *(*Transforming growth factor alpha*) (g.3.11.1.8). For each method we report the top 16 neighbors and their distance/similarity. To highlight relations within families and superfamilies we represent each SCOP domain by its family designation and append a serial number to create a unique identifier. The complete list of SCOP IDs and their numeric designations is available at

**BLAST**		**PSIBLAST**		**FUGUE**		***DP-PSIBLAST***		***DP-FUGUE***	
g.3.11.1.8	1e-18	g.3.11.1.8	1e-19	g.3.11.1.8	26.970	g.3.11.1.8	0.0000	g.3.11.1.8	-
g.3.11.1.9	8e-04	g.3.11.1.9	3e-04	g.3.11.1.9	9.830	g.3.11.1.9	0.2066	g.3.11.1.10	101.76
g.3.11.1.10	0.067	g.3.11.1.10	0.028	g.3.11.1.10	9.390	g.3.11.1.7	0.4665	g.3.11.1.9	96.05
g.3.11.1.7	0.11	g.3.11.1.7	0.057	g.3.11.1.5	5.920	g.3.11.1.10	0.5266	g.3.11.1.3	88.64
**d.158.1.1.1**	**1.9**	**d.158.1.1.1**	**1.2**	g.3.11.1.7	5.430	g.3.11.1.3	1.2862	g.3.11.1.16	75.32
c.69.1.19.1	4.6	c.69.1.19.1	2.0	g.3.11.1.3	5.350	g.3.11.1.1	1.6956	g.3.11.1.1	74.53
b.40.2.1.5	7.3	b.40.2.1.5	6.3	g.3.11.1.16	5.310	**d.158.1.1.1**	**1.8567**	g.3.11.1.7	73.60
d.159.1.3.3	7.9	d.159.1.3.3	8.3	**g.27.1.1.5**	**5.050**	a.102.1.1.2	1.8617	g.3.11.1.14	70.52
a.118.8.1.5	11	a.118.8.1.5	12	g.3.11.1.14	4.760	d.10.1.3.2	1.8617	g.3.11.1.5	68.21
b.77.3.1.2	17	b.68.1.1.2	14	g.3.11.1.1	4.040	d.13.1.1.2	1.8617	g.3.11.1.6	61.55
b.68.1.1.2	21	b.29.1.3.1	15	a.4.10.1.1	3.990	d.15.9.1.1	1.8617	**g.27.1.1.4**	**37.84**
c.1.10.2.1	21	a.138.1.3.1	16	d.158.1.1.1	3.870	d.5.1.1.2	1.8617	g.3.11.1.13	35.63
b.45.1.2.1	23	b.77.3.1.2	17	g.3.11.1.2	3.790	a.45.1.1.1	1.8719	g.3.11.1.18	30.36
b.29.1.3.1	24	c.1.10.2.1	22	g.3.11.1.6	3.600	d.169.1.2.1	1.8987	g.3.11.1.17	28.75
d.58.3.1.2	25	b.45.1.2.1	24	g.26.1.1.1	3.570	c.3.1.2.7	1.9085	g.3.11.1.11	25.26

**Table 11:** Closest neighbors of *d*1*hdoa_ *(*Biliverdin IX beta reductase*) (c.2.1.2.26). For each method we report the top 33 neighbors and their distance/similarity.

**BLAST**		**PSIBLAST**		**FUGUE**		***DP-PSIBLAST***		***DP-FUGUE***	
c.2.1.2.26	1e-110	c.2.1.2.26	1e-120	c.2.1.2.26	62.720	c.2.1.2.26	0.0000	c.2.1.2.26	-
c.2.1.2.1	0.12	c.2.1.2.1	0.069	c.2.1.2.27	6.310	c.2.1.2.4	1.4380	c.2.1.2.14	102.46
**c.69.1.1.3**	**0.73**	**c.69.1.1.3**	**0.34**	c.2.1.3.5	6.270	**d.108.1.1.1**	**1.5288**	c.2.1.2.13	91.26
c.31.1.5.1	1.3	d.108.1.1.1	0.65	c.2.1.2.13	6.250	b.7.1.1.5	1.6281	c.2.1.2.18	90.81
d.108.1.1.1	1.3	c.31.1.5.1	1.1	c.2.1.2.7	5.810	b.82.2.2.1	1.6281	c.2.1.2.17	85.56
a.93.1.1.4	2.4	a.93.1.1.4	2.2	**c.78.2.1.1**	**5.620**	c.81.1.1.2	1.6326	c.2.1.2.23	84.11
c.1.2.4.6	3.7	c.4.1.2.2	2.5	c.2.1.2.16	5.570	c.2.1.2.3	1.6598	c.2.1.2.15	84.01
d.144.1.1.12	3.9	c.1.2.4.6	2.6	c.2.1.3.6	4.930	c.66.1.13.1	1.6834	c.2.1.2.7	80.02
d.127.1.1.4	5.2	d.144.1.1.12	3.6	c.23.5.1.1	4.740	c.2.1.2.5	1.6908	c.2.1.2.25	80.00
c.4.1.2.2	5.3	d.127.1.1.4	4.2	c.2.1.6.5	4.520	c.2.1.2.1	1.6957	c.2.1.2.19	73.76
c.37.1.8.1	6.1	c.37.1.8.1	4.8	c.2.1.6.6	4.410	c.69.1.1.1	1.7249	c.2.1.2.11	73.52
b.77.2.1.1	7.4	a.118.2.1.5	6.2	d.142.1.2.3	4.370	c.1.8.7.1	1.7272	c.2.1.2.8	72.24
c.3.1.2.7	7.9	a.104.1.1.4	6.3	c.2.1.2.9	4.330	c.55.7.1.3	1.7273	c.2.1.2.21	71.62
a.118.2.1.5	8.3	b.77.2.1.1	6.4	c.34.1.1.1	4.210	d.108.1.1.2	1.7273	c.2.1.2.16	69.59
d.126.1.3.1	10	a.79.1.1.1	6.7	c.4.1.2.2	4.200	c.69.1.1.2	1.7277	c.2.1.2.10	69.55
c.1.10.1.4	12	c.3.1.2.7	6.8	c.93.1.1.1	4.080	c.69.1.17.4	1.7374	c.2.1.6.12	69.55
a.104.1.1.4	15	c.60.1.3.1	7.5	c.2.1.5.2	3.970	c.69.1.2.1	1.7412	c.2.1.2.20	69.55
c.60.1.3.1	15	c.59.1.1.3	7.7	c.2.1.2.15	3.940	c.2.1.2.2	1.7443	c.2.1.2.24	69.55
a.56.1.1.3	16	c.1.10.1.4	7.8	c.3.1.2.2	3.910	c.69.1.2.2	1.7502	c.2.1.2.27	69.55
d.104.1.1.13	17	a.118.2.1.2	7.9	c.37.1.2.1	3.900	d.108.1.1.4	1.7548	c.2.1.2.9	69.16
d.15.9.1.1	18	d.126.1.3.1	8.0	c.37.1.8.4	3.890	b.93.1.1.1	1.7658	c.2.1.2.28	68.02
a.79.1.1.1	19	c.37.1.10.7	8.8	c.2.1.2.19	3.850	c.2.1.2.6	1.7731	c.2.1.2.2	63.80
b.60.1.1.7	19	c.31.1.3.3	8.9	a.146.1.1.1	3.820	e.6.1.1.2	1.7799	c.2.1.2.22	61.33
c.45.1.2.4	19	d.104.1.1.13	9.4	c.2.1.2.28	3.820	b.40.5.1.2	1.8001	c.2.1.6.3	51.80
b.1.1.5.24	20	c.45.1.2.4	11	c.3.1.5.14	3.770	c.69.1.1.4	1.8074	c.2.1.6.6	48.27
c.31.1.3.3	20	b.43.3.2.1	12	c.2.1.2.1	3.760	c.69.1.1.3	1.8135	c.2.1.5.7	45.83
e.28.1.1.1	20	c.93.1.1.10	12	c.2.1.2.17	3.750	c.78.1.1.6	1.8210	c.2.1.2.1	45.75
b.1.1.1.5	22	b.1.1.5.24	13	c.2.1.3.7	3.740	g.17.1.3.1	1.8259	c.2.1.5.9	42.88
c.59.1.1.3	22	d.15.9.1.1	13	d.95.1.1.1	3.700	a.138.1.3.5	1.8261	**c.4.1.2.2**	**37.63**
c.93.1.1.10	22	c.29.1.1.2	14	g.18.1.1.12	3.700	a.144.1.1.2	1.8261	c.3.1.2.3	37.57
e.29.1.1.1	22	c.93.1.1.1	14	c.2.1.9.1	3.680	c.31.1.2.1	1.8261	c.3.1.4.1	37.09
b.60.1.1.4	23	e.29.1.1.1	14	c.68.1.3.1	3.650	c.31.1.5.1	1.8261	c.2.1.3.	36.66
.		.		.		.		.	
.		.		.		.		.	
.		.		.		.		.	

## References

[B1] Biozon Database Release 2.0. http://biozon.org/.

[B2] Pearson WR (1997). Identifying distantly related protein sequences. Comp Appl Biosci.

[B3] Brenner SE, Chothia C, Hubbard TJP (1998). Assessing sequence comparison methods with reliable structurally identified distant evolutionary relationships. Proc Natl Acad Sci USA.

[B4] Sippl MJ (1990). Calculation of conformational ensembles from potentials of mean force. An approach to the knowledge-based prediction of local structures in globular proteins. J Mol Biol.

[B5] Altschul SF, Madden TL, Schäffer AA, Zhang J, Zhang Z, Miller W, Lipman DJ (1997). Gapped BLAST and PSI-BLAST: a new generation of protein database search programs. Nucleic Acids Res.

[B6] Karplus K, Barrett C, Cline M, Diekhans M, Grate L, Hughey R (1999). Predicting protein structure using only sequence information. Proteins.

[B7] Yona G, Levitt M (2002). Within the twilight zone: a sensitive profile-profile comparison tool based on information theory. J Mol Biol.

[B8] Sadreyev R, Grishin N (2003). COMPASS: a tool for comparison of multiple protein alignments with assessment of statistical significance. J Mol Biol.

[B9] van Heel M (1991). A new family of powerful multivariate statistical sequence analysis techniques. J Mol Biol.

[B10] Ferran EA, Pflugfelder B, Ferrara P (1994). Self-Organized Neural Maps of Human Protein Sequences. Protein Sci.

[B11] Hobohm U, Sander C (1995). A sequence property approach to searching protein database. J Mol Biol.

[B12] Wu C, Whitson G, Mclarty J, Ermongkonchai A, Chang T (1992). Protein classification artificial neural system. Protein Sci.

[B13] Jaakkola T, Diekhans M, Haussler D (1999). Using the Fisher kernel method to detect remote protein homologies. Proc Int Conf Intell Syst Mol Biol.

[B14] Tsuda K, Kin T, Asai K (2002). Marginalized kernels for biological sequences. Bioinformatics.

[B15] Leslie C, Eskin E, Noble WS (2002). The spectrum kernel: a string kernel for SVM protein classification. Pac Symp Biocomput.

[B16] Leslie CS, Eskin E, Cohen A, Weston J, Noble WS (2002). Mismatch string kernels for discriminative protein classification. Bioinformatics.

[B17] Eskin E, Snir S (2004). The homology kernel: a biologically motivated sequence embedding into Euclidean space. Technical report.

[B18] Seeger M (2002). Covariance kernels from Bayesian generative models. Neural Inf Proc Syst.

[B19] Cuturi M, Vert J-P (2004). A mutual informatio kernel for sequences. Proceedings of the International Joint Conference on Neural Networks.

[B20] Ron D, Singer Y, Tishby N (1996). The power of amnesia: learning probabilistic automata with variable memory length. Machine Learning.

[B21] Liao L, Noble WS (2003). Combining pairwise sequence similarity and support vector machines for detecting remote protein evolutionary and structural relationship. J Comp Biol.

[B22] Pereira F, Tishby N, Lee L Distributional Clustering of English Words. Proceedings of the 31st Annual Meeting of the Association for Computational Linguistics, 22–26 June 1993.

[B23] Joachims T, Cristianini N, Shawe-Taylor J Composite Kernels for Hypertext Categorisation. Proceedings of the International Conference on Machine Learning, Williams College, June 28–July 1, 2001.

[B24] Karplus K, Karchin R, Barrett C, Tu S, Cline M, Diekhans M, Grate L, Casper J, Hughey R (2001). What is the value added by human intervention in protein structure prediction?. Proteins.

[B25] Dubnov S, El-Yaniv R, Gdalyahu Y, Schneidman E, Tishby N, Yona G (2002). A new nonparametric pairwise clustering algorithm based on iterative estimation of distance profiles. Machine Learning.

[B26] Yona G, Levitt M (2000). Towards a complete map of the protein space based on a unified sequence and structure analysis of all known proteins. Proc Int Conf Intell Syst Mol Biol.

[B27] Murzin AG, Brenner SE, Hubbard T, Chothia C (1995). SCOP: a structural classification of proteins database for the investigation of sequences and structures. J Mol Biol.

[B28] Chandonia JM, Hon G, Walker NS, Lo Conte L, Koehl P, Levitt M, Brenner SE (2004). The ASTRAL compendium in 2004. Nucleic Acids Res.

[B29] Berman HM, Westbrook J, Feng Z, Gilliland G, Bhat TN, Weissig H, Shindyalov IN, Bourne PE (2000). The Protein Data Bank. Nucleic Acids Res.

[B30] Shi J, Blundell TL, Mizuguchi K (2001). FUGUE: sequence-structure homology recognition using environment-specific substitution tables and structure-dependent gap penalties. J Mol Biol.

[B31] Pearson WR (1998). Empirical statistical estimates for sequence similarity searches. J Mol Biol.

[B32] Karplus K, Barrett C, Hughey R (1998). Hidden Markov models for detecting remote protein homologies. Bioinformatics.

[B33] Eddy SR (1998). Profile hidden Markov models. Bioinformatics.

[B34] Lin J (1991). Divergence measures based on the Shannon entropy. IEEE Trans on Information Theory.

[B35] Kullback S (1959). Information Theory and Statistics.

[B36] Chung R, Yona G (2004). Protein family comparison using statistical models and predicted structural information. BMC Bioinformatics.

[B37] Edgar RC, Sjolander K (2004). A comparison of scoring functions for protein sequence profile alignment. Bioinformatics.

[B38] Nagarajan N, Yona G (2004). Automatic prediction of protein domains from sequence information using a hybrid learning system. Bioinformatics.

[B39] Gribskov M, Mclachlen AD, Eisenberg D (1987). Profile analysis: detection of distantly related proteins. Proc Natl Acad Sci USA.

[B40] Lindahl E, Elofsson A (2000). Identification of related proteins on family, superfamily and fold level. J Mol Biol.

[B41] Egan JP (1975). Signal Detection Theory and ROC Analysis.

[B42] Altschul SF, Gish W, Miller W, Myers EW, Lipman DJ (1990). Basic local alignment search tool. J Mol Biol.

[B43] Yona G, Kedem K (2005). The URMS-RMS hybrid algorithm for fast and sensitive local protein structure alignment. J Comp Biol.

[B44] Altschul SF, Gish W (1996). Local alignment statistics. Methods Enzymol.

[B45] Park J, Karplus K, Barrett C, Hughey R, Haussler D, Hubbard T, Chothia C (1998). Sequence comparisons using multiple seuqences detect three times as many remote homologues as pairwise methods. J Mol Biol.

[B46] Flannick J, Batzoglou S (2005). Using multiple alignments to improve seeded local alignment algorithms. Nucleic Acids Res.

[B47] Thompson JD, Higgins DG, Gibson TJ (1994). CLUSTAL W: improving the sensitivity of progressive multiple sequence alignment through sequence weighting, position-specific gap penalties and weight matrix choice. Nucleic Acids Res.

[B48] Valentini G, Masulli F, Marinaro M, Tagliaferri R Ensembles of learning machines. Neural Nets WIRN Vietri-2002, Series Lecture Notes in Computer Sciences.

[B49] Sauder JM, Arthur JW, Dunbrack RL (2000). Large-scale comparison of protein sequence alignment algorithms with structure alignments. Proteins.

[B50] Data used for the distance-profile representation. http://biozon.org/ftp/data/papers/distance-profile/.

[B51] Jones DT, Taylor WR, Thorton JM (1992). A new approach to protein fold recognition. Nature.

[B52] Huber T, Torda AE (1999). Protein sequence threading, the alignment problem, and a two-step strategy. J Comput Chem.

[B53] Jones DT (1999). GenTHREADER: an efficient and reliable protein fold recognition method for genomic sequences. J Mol Biol.

[B54] Russell AJ, Torda AE (2002). Protein sequence threading: averaging over structures. Proteins.

